# Metagenomic characterization of the tracheobronchial microbiome in lung cancer

**DOI:** 10.3389/frmbi.2024.1457537

**Published:** 2024-11-21

**Authors:** Alexis Bailey, Stephanie Hogue, Christine M. Pierce, Shirlene Paul, Natalie La Fuente, Ram Thapa, Youngchul Kim, Lary A. Robinson

**Affiliations:** ^1^ Division of Thoracic Oncology, Moffitt Cancer Center, Tampa, FL, United States; ^2^ Department of Cancer Epidemiology, Moffitt Cancer Center, Tampa, FL, United States; ^3^ Department of Biostatistics and Bioinformatics, Moffitt Cancer Center, Tampa, FL, United States

**Keywords:** lung cancer microbiome, non-small cell lung cancer, tracheal microbiota, oral microbiota, lung microbiome, microaspiration, lung inflammation

## Abstract

**Background:**

The tracheobronchial and oral microbiome may be associated with lung cancer, potentially acting as predictive biomarkers. Therefore, we studied the lung and oral bacteriome and virome in non-small cell lung cancer (NSCLC) patients compared to melanoma controls to discover distinguishable features that may suggest lung cancer microbial biomarkers.

**Methods:**

In this pilot case-control study, we recruited ten patients with early-stage NSCLC (cases) and ten age-matched melanoma patients (controls) who both underwent tumor resection. Preoperative oral gargles were collected from both groups, who then underwent transbronchoscopic tracheal lavage after intubation. Lung tumor and adjacent non-neoplastic lung were sterilely collected after resection. Microbial DNA from all lung specimens underwent 16S rRNA gene sequencing. Lavage and gargle specimens underwent whole-genome shotgun sequencing. Microbiome metrics were calculated to compare both cohorts. T-tests and Wilcoxon rank sum tests were used to test for significant differences in alpha diversity between cohorts. PERMANOVA was used to compare beta diversity.

**Results:**

No clear differences were found in the microbial community structure of case and control gargles, but beta diversity of case and control lavages significantly differed. Two species, *Granulicatella adiacens* and *Neisseria subflava*, which are both common oral commensal organisms, appeared in much higher abundance in case versus control lavages. Case lavages also maintained higher relative abundances of other oral commensals compared to controls.

**Conclusions:**

Lung lavages demonstrated oral microbiota enrichment in cases compared to controls, suggesting microaspiration and resultant inflammation. The oral commensals *Granulicatella adiacens* and *Neisseria subflava* were more abundant in the tracheobronchial lavages of lung cancer versus melanoma patients, implicating these microorganisms as potential lung cancer biomarkers, warranting further validation studies.

## 1 Introduction

Lung cancer is the most common cancer worldwide and the leading cause of cancer deaths, with 1.8 million deaths in 2020 (World Health Organization, 2022). In the US, lung cancer has the second highest incidence rate among both males and females, but it is the most common cause of death among both sexes (Henley et al., 2020). An estimated 130,450 Americans will die from lung cancer in 2024, exceeding the number of deaths expected from colon, breast, and prostate cancers combined (American Cancer Society, 2024). Despite enormous research and treatment efforts, the high fatality rate of this malignancy (75%) has changed little over the last few decades (American Lung Association, 2024). The high mortality rate of lung cancer is primarily due to delayed diagnosis, with 77% of cases not being recognized until later stages (Mao et al., 2016). Screening low dose chest computed tomography is underutilized, with only 5.5% of eligible individuals obtaining a scan despite its proven potential to detect early-stage diseases in high-risk individuals (Richards et al., 2019). Therefore, exploration of potential biomarkers of this disease is warranted.

As the affordability of next-generation sequencing techniques improves, the microbiome, or the collective genomic material of all microorganisms found within and on the body, is increasingly being investigated for associations with disease and potential therapeutic value. Most research has focused on the gut microbiome, the largest and most diverse microbiome in the human body, with relatively little investigation of the microbiota of other anatomic sites. Until recently, the lungs were considered sterile, but evidence indicates commensal microbes, including Acinetobacter, Pseudomonas, and Ralstonia, indeed colonize this organ (Yu et al., 2016). Furthermore, the composition and function of the microbiota in lung tissue are distinct from other anatomic sites, including the oral cavity (Yu et al., 2016).

Recent research has further shown associations between the local lung microbiome and various lung pathologies, such as asthma, cystic fibrosis, and chronic obstructive pulmonary disease (COPD) (Moffatt and Cookson, 2017; Mur et al., 2018). Additionally, hypotheses regarding an association between the lung microbiome and lung cancer, potentially mediated by chronic inflammation, have been suggested (Mur et al., 2018). Nevertheless, relatively little research on the lung microbiome in the context of lung cancer has been conducted.

Given the potential for the lung microbiome to be associated with lung cancer and to be utilized as a biomarker, this study aimed to characterize lung and oral bacteriomes and viromes in early-stage non-small cell lung cancer (NSCLC) patients compared to melanoma controls to identify distinguishable features in their oral, tracheal, and tumor microbiomes that may suggest a reliable microbial biomarker for the presence of lung cancer. The results may help assess the potential of minimally invasive samples from the oral flora that might act as proxies for tumor microbiomes, potentially leading to the development of a reliable screening technique for high-risk individuals with early cancers. While the presence of specific microorganisms found in conjunction with lung cancer is intriguing, this study is not focused on correlating these findings with possible lung carcinogenesis.

## 2 Methods

### 2.1 Patients

This prospective, exploratory case-control study recruited ten early-stage NSCLC patients and ten control melanoma patients undergoing surgical resection of their tumor under general anesthesia at Moffitt Cancer Center between July 2015 and May 2016. Melanoma patients were chosen as the best possible proxy for controls because this cancer type has not shown clear evidence of microbial etiology (Woo et al., 2022) that would alter the normal oral and respiratory flora, and these patients were already undergoing anesthesia with intubation for major resection of locally-advanced peripheral melanomas on extremities, far distant from the lung. Theoretically, the best control would be bronchoscopy and tracheobronchial lavages on normal people without cancer or lung disease. However, it would be exceedingly difficult to recruit these individuals and we had ethical concerns about recommending this procedure with its inherent risks for this exploratory study. In general, the usual reasons for performing bronchoscopy on patients without diagnosed cancer are either for evaluation of lung infections and/or to biopsy lung abnormalities which might be cancer. Obviously, these patients would not be valid controls.

Lung cancer cases and melanoma controls were matched by age (± 10 years) and smoking status (current/former versus never-smokers). Eligible participants were at least 21 years of age, mentally competent, not pregnant, and received no chemotherapy within 1 year of surgery. Furthermore, participants could not have post-obstructive pneumonitis, current pneumonitis, purulent bronchitis, other acute respiratory infections, cystic fibrosis, clinically significant bronchiectasis, other inflammatory or fibrotic lung diseases, chronic or current corticosteroid use, antimicrobial therapy within 1 month or prebiotics/probiotics within 3 months of surgery. We performed this study per the ethical standards established in the 1964 Declaration of Helsinki and its later amendments. It was approved by the Liberty Institutional Review Board, Protocol 14.12.0036 (MCC 17976). We obtained informed consent from all participants.

### 2.2 Specimen collection

#### 2.2.1 Tracheobronchial lavages

We collected intraoperative tracheobronchial lavages in all patients. After induction of general anesthesia and within two minutes of endotracheal intubation with a sterile single-use tube, LAR performed bronchoscopy with tracheal lavage using 50-100mL of sterile 0.9% normal saline solution and an Olympus pediatric bronchoscope pre-cleaned and disinfected with Steris (Steris System 1E Liquid Chemical Sterilant Processing System, Steris Corporation, Mentor, OH) according to CDC guidelines (Rutala and Weber, 2008). An intravenous, preoperative, prophylactic antibiotic was started during bronchoscopy, so it would not have reached a therapeutic blood level when we obtained lavage samples. We collected approximately 20 mL of tracheobronchial lavage fluid into a sterile Lukens trap (Argyle™ Specimen Trap, Cardinal Health Inc., Dublin, OH), transported on ice to the laboratory, and processed by centrifugation at 3,000 x g for 15 minutes at 4°C to separate supernatant and cell pellet. 3.2mL of supernatant was pipetted between two cryovials. Cell pellets were re-suspended in 1.2mL of sterile PBS and aliquoted evenly between two cryovials, which were snap-frozen in liquid nitrogen. Cell pellets were snap frozen in liquid nitrogen (LN) and stored at -80°C.

#### 2.2.2 Oral gargle samples

We also collected oral gargles from cases and controls in the preoperative area. Participants vigorously swished and gargled 15mL of disinfectant-free mouthwash for 15 seconds, then expectorated into a sterile 50mL conical tube. Specimens were centrifuged according to the same parameters as lavages. We collected 3.2mL of supernatant between two cryovials. The cell pellet was re-suspended in 20mL of PBS and centrifuged again at the same speed, duration, and temperature. The final cell pellet was re-suspended in 1.2mL PBS and aliquoted as two 0.6mL aliquots stored at -80°C.

#### 2.2.3 Tissue samples

Only lung cancer patients provided tumor and adjacent non-neoplastic lung tissue specimens. Immediately after resection, the surgeon (LAR, the thoracic surgeon on all cases), while wearing a mask, took the resected specimen in a sterile container to the frozen section room and after the pathologist removed specimens needed for clinical pathology (diagnosis, margins, and lymph nodes), LAR removed 1cm^3^ from the tumor using sterile gloves and instruments in a sterile field. LAR also harvested a similar-sized, non-neoplastic lung specimen in the same manner at a distance from the tumor. Tissue specimens were transported to the laboratory and snap frozen in LN before undergoing macrodissection and long-term storage at -80°C.

### 2.3 DNA extraction

We extracted microbial DNA from all sample types. The MoBio^®^ PowerSoil DNA isolation kit (Qiagen, Germantown, MD) was utilized in a modified protocol to extract bacterial DNA from 0.6mL cell pellets from lavages and gargles. Briefly, cell pellets were vortexed and spun down until the sample collected at the bottom of the tube. It was then added to a bead-beating tube with buffer and processed in the MP-Bio Fastprep™ 5G (MP Biomedicals, Irvine, CA) for 30 seconds at 6m/s for each of 2 cycles. Samples were centrifuged at 10,000 x g for 30 seconds at room temperature with the resulting supernatant collected. The supernatant was processed to remove PCR inhibitors and eluted with 100µL of buffer. DNA was quantitated using Qubit, and quality was checked using Nanodrop.

We used the Qiagen^®^ DNeasy Blood and Tissue kit (Qiagen, Germantown, MD) to isolate DNA from tissue samples according to the manufacturer’s protocol. Approximately 25mg was utilized, or about half of the total tissue volume. We briefly added the tissue to a bead-beating tube containing 360 µL of ATL buffer and 40 µL of proteinase K before being vortexed and incubated in the lytic step. Samples were bead-beat according to the same steps outlined above. Samples were then centrifuged at 20,000xg for 3 minutes, and the resulting supernatant was further processed and eluted in buffer AE.

### 2.4 16S rRNA gene sequencing

All samples underwent 16S rRNA gene sequencing with appropriate controls. Libraries were prepared using standard operating procedures (SOPs) from the Weinstock Lab at the Jackson Laboratory (The Jackson Laboratory, Farmington, CT). Briefly, high-performance liquid chromatography-purified primers and 4ng of DNA template were used to amplify the V1-V3 regions of the 16S rRNA gene. Libraries were screened for size and quantity as described in the SOP, and after pooling, they were quantified by qPCR using the Kapa Library Quantification Kit. The final libraries were sequenced with a 50% PhiX spike-in on an Illumina MiSeq v3 2x300 sequencing run. The raw reads were submitted to the NCBI Short Read Archive (SRA) under BioProject PRJNA1177881.

### 2.5 Metagenomic whole genome shotgun sequencing

DNA was isolated from all oral gargles and lung lavages. Whole genome shotgun DNA libraries were prepared from 100ng of DNA using the Illumina TruSeq Nano DNA kit following the manufacturer’s protocol (Illumina, Inc., San Diego, CA). The libraries were sequenced on Illumina NextSeq High Output Kits v2 2x150 to about 80 to 260 million paired-end reads, depending on the percent alignment to microbial species. This method was utilized to resolve bacterial signatures to the species level and to identify viral signatures. Fungal sequences were not examined in the WGSS results.

### 2.6 Bioinformatics and statistical analyses

#### 2.6.1 16s rRNA sequencing data analysis

Paired-end sequencing reads were cleaned using Trimmomatic v. 0.39 (Bolger et al., 2014) with the following parameters: LEADING:3 TRAILING:3 SLIDINGWINDOW:4:15 MINLEN:36 to remove adaptors and low-quality reads. Treatment samples with a minimum of 2,000 reads were kept for further downstream analysis. The chimeric reads were searched against the 16S rRNA Gold database with the default UCHIME (4.2) parameters (Edgar, 2016). Next, the cleaned reads were merged with PEAR (0.9.10) (Zhang et al., 2014) and operational taxonomic units (OTUs) were generated by open reference of QIIME1.9.1 pipeline (Caporaso et al., 2010). Only OTUs with a minimum observation count of 100 were retained. The database used for the taxonomic assignment was Silva 128 97_otus_16S.fasta (Quast et al., 2013). Alpha- and Beta-diversity were analyzed using QIIME1.9.1. The taxonomy plots were based on the 25 most prevalent OTUs. PERMANOVA was used to compare beta diversity estimates.

Fold differences in the top 25 most abundant microbes, with relative abundances of at least 1% in one comparison group, were calculated by dividing the relative abundance of the microbe in the comparison groups. Similarly, fold differences in the top 25 most prevalent microbes with prevalence of at least 10% in either comparison group were calculated and organized into Venn diagrams. Student two-sample t-test and Wilcoxon rank sum test were used for differential abundance analysis between cases, controls, and sample types. Two-sided P values <0.05 were considered statistically significant. Statistical analysis was completed using the Phyloseq package in R software (v3.1.1 and v4.1.0, The R Foundation, Vienna, Austria).

#### 2.6.2 WGSS data processing analysis for taxonomic classification methods

The CosmosID platform was used to process WGSS data and perform strain-level taxonomic classification. Briefly, their algorithm disambiguated short sequence reads into discrete genomes. The pipeline used pre-computation phases [using the CosmosID taxonomic reference databases containing bacteria, viruses, phages, virulence markers, and antimicrobial resistance markers curated by CosmosID (CosmosID, Inc., Germantown, MD) (CosmosID Inc, 2021)] with per-sample computation (searches short sequence reads or contigs from draft *de novo* assemblies against fingerprint sets), detect and classify microbial sequencing reads. The platform filtered reads using a filtering threshold derived from internal scores determined by analyzing many diverse metagenomes to exclude false positives.

## 3 Results

### 3.1 Patient characteristics

All 20 participants (ten NSCLC cases and ten melanoma controls) were Caucasian (**Table 1**). Cases had a higher percentage of females than controls (40% vs. 20%). Most lung cancer patients had stage I disease (80%), while most melanoma controls were advanced stage (50% had stage III disease). Most cases (90%) and controls (80%) had not received antibiotics within 2 months before their surgery. No significant differences were observed in the characteristics measured between cases and controls.

**Table 1:** Distribution of sample characteristics by lung cancer cases (n=10) versus melanoma control (n=10) status.

Characteristic	Categories	Cases, No. (%)	Controls, No. (%)	*p* value
**Age, mean ± SD**	–	71.2 ± 9.3	71.8 ± 12.0	0.791
**Sex**	Male	6 (60.0)	8 (80.0)	0.628
	Female	4 (40.0)	2 (20.0)	
**Race**	White	10 (100.0)	10 (100.0)	N/A
	Non-white	0 (0.0)	0 (0.0)	
**Ethnicity**	Hispanic	0 (0.0)	1 (10.0)	1.000
	Non-Hispanic	10 (100.0)	9 (90.0)	
**Marital status**	Married	7 (70.0)	7 (70.0)	1.000
	Divorced/separated	1 (10.0)	1 (10.0)	
	Widowed	1 (10.0)	2 (20.0)	
	Single	1 (10.0)	0 (0.0)	
**Stage**	I IA IB	8 (80.0) 3 (30.0) 5 (50.0)	3 (3.0) 2 (20.0) 1 (10.0)	0.212
	II IIA IIC	1 (10.0) 1 (10.0) 0 (0.0)	1 (10.0) 0 (0.0) 1 (10.0)	
	III IIIA IIIB IIIC	1 (10.0) 1 (10.0) 0 (0.0) 0 (0.0)	5 (50.0) 2 (20.0) 1 (10.0) 2 (20.0)	
	Not staged	0 (0.0)	1 (10.0)	
**Antibiotic use**	< 2 Mo. before surgery	1 (10.0)	2 (20.0)	1.000
	> 2 Mo. before surgery	9 (90.0)	8 (80.0)	
**Smoking status**	Current/former smokers	5 (50.0)	5 (50.0)	N/A
	Never smokers	5 (50.0)	5 (50.0)	

Mo., months; N/A, not applicable; SD, standard deviation. Fisher’s exact test and Wilcoxon rank sum test were used to determine if distributions of categorical and continuous variables differed according to case or control status, respectively.

### 3.2 Microbial profiling

#### 3.2.1 Cases vs. controls: tracheobronchial lavages

##### 3.2.1.1 16S rRNA gene sequencing

**Figure 1:**
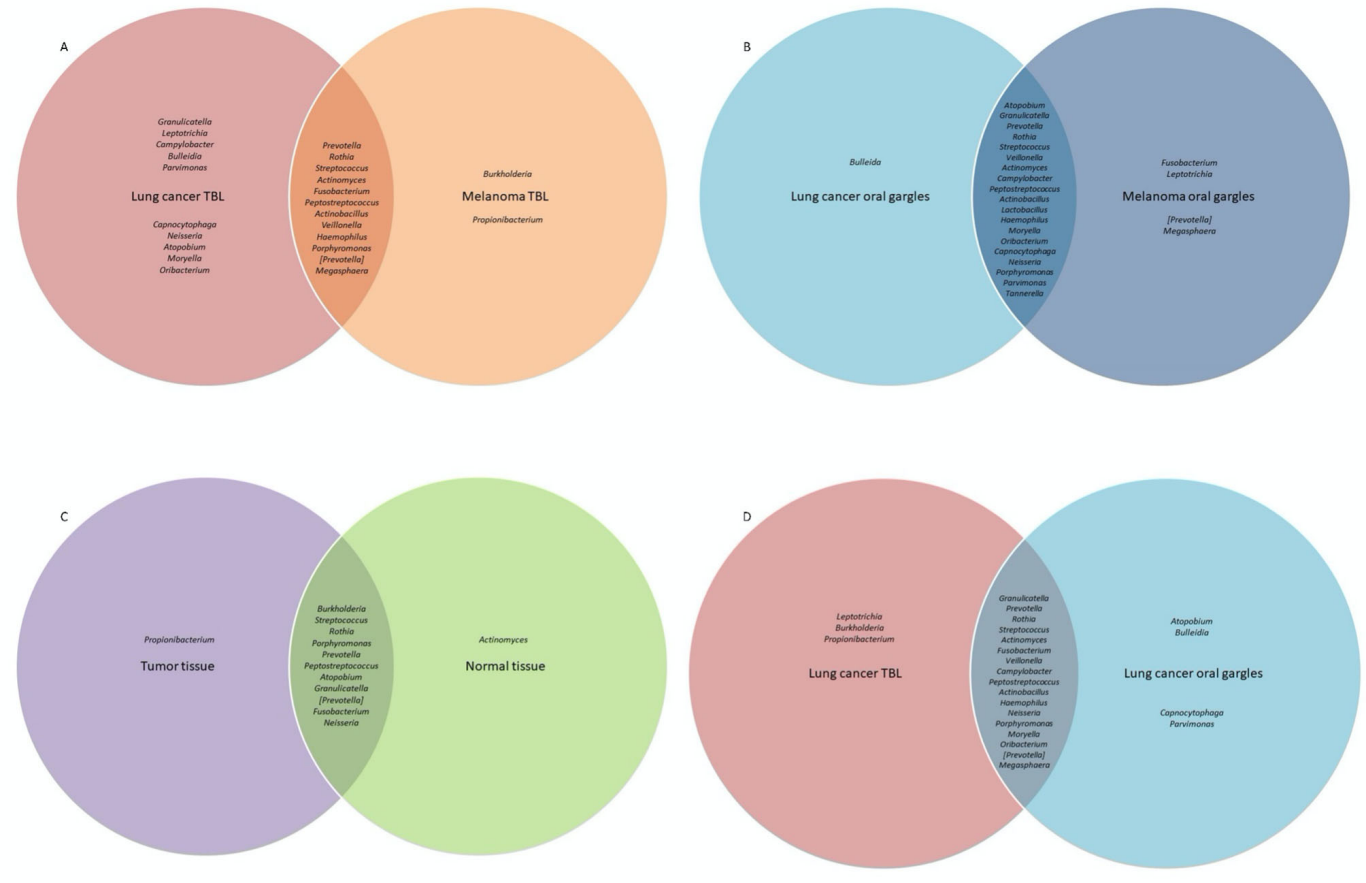
Venn diagram comparison of bacterial genera prevalence between cases and controls and between different sample types among controls, as determined by the top 25 most prevalent bacterial genera identified in 16S rRNA sequencing analyses. Bacterial genera in the center of the Venn diagram are found in both groups. **(A)** Comparison of bacterial prevalence between lung cancer and melanoma tracheobronchial lavages (TBL). **(B)** Comparison of bacterial prevalence between lung cancer and melanoma oral gargles. **(C)** Comparison of bacterial prevalence between tumor and normal tissue from lung cancer cases. **(D)** Comparison of bacterial prevalence between tracheobronchial lavages and oral gargles from lung cancer cases.

**Figure 2:**
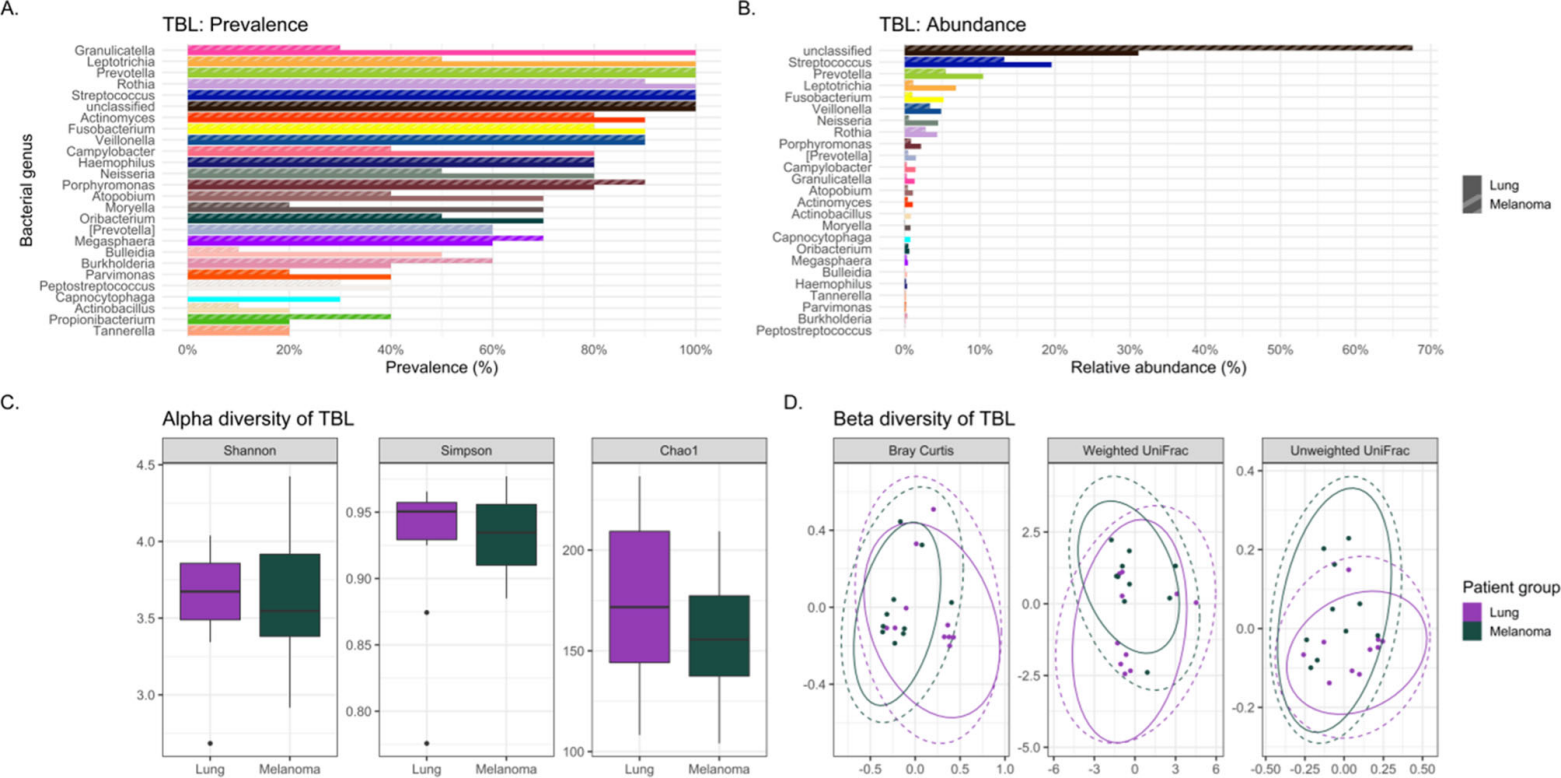
Comparing the bacteriomes, assessed by 16S rRNA gene sequencing, of tracheobronchial lavages (TBLs) of lung cancer cases and melanoma controls. **(A)** Comparison of the prevalence of bacterial genera in lung cancer versus melanoma control TBLs. **(B)** Comparison of the relative abundance of bacterial genera in lung cancer versus melanoma control TBLs. **(C)** Comparison of the alpha diversity, as measured by Shannon, Simpson, and Chao1 indices, between lung cancer case and melanoma control TBLs. **(D)** Comparison of the beta diversity, measured by Bray Curtis, Weighted and Unweighted UniFrac distance measures, between lung cancer case and melanoma control TBLs.

The usual lower airway genera *Streptococcus and Prevotella (* CosmosID Inc, 2021) were identified in all lavages (**Figure 1A**). However, the oral commensals appeared more prevalent among cases versus controls: *Granulicatella (100% versus 30%), Leptotrichia (100% versus 50%), Moryella (70% versus 20%)*, and *Neisseria (80% versus 50%)* respectively (**Figure 2A**). *Neisseria* was nearly eight-fold more abundant in tracheobronchial lavages of cases versus controls (**Table 2**; **Figure 2B**) (Lee et al., 2016; Huffnagle et al., 2017).

**Table 2:** Relative abundance comparisons, using fold changes, of the top 25 most abundant (for taxa in >1% abundance) bacterial genera bacterial species, and viral taxa between case and control specimens and case tissue specimens.

Comparison		Sequencing Methodology		
		16S rRNA gene sequencing	WGSS (bacterial)	WGSS (viral)
**Lavages: Lung cancer compared to melanoma**	Higher abundance in Lung cancer	1. ***Neisseria*** (7.82x) 2. ***Leptotrichia*** (5.94x) 3. *Campylobacter* (5.37x) 4. ***Fusobacterium*** (5.02x) 5. ***Granulicatella*** (4.55x) 6. **[*Prevotella*]** (3.10) 7. ***Porphyromonas*** and *Actinomyces* (2.60x) 8. ***Atopobium*** (2.50x) 9. ***Prevotella*** (1.91x) 10. ***Rothia*** (1.55x) 11. ***Streptococcus*** (1.47x)	1. ***Gemella haemolysans*** (26.17x) 2. ***Neisseria subflava*** (>15.93x) 3. ***Porphyromonas* KLE1280** 13.56x) 4. ***Granulicatella adiacens*** (>6.18x) 5. ***Rothia dentocariosa*** (1.80x) 6. ***Rothia mucilaginosa*** (1.39x)	1. Human betaherpesvirus 7 (>22.1x)
	Lower abundance in Lung Cancer	N/A	1. *Megasphaera micronuciformis* (0.06x) 2. ***Prevotella histicola*** (0.07x) 3. *Veillonella dispar* (0.13x) 4. ***Prevotella pallens*** (0.18x)	1. Human respiratory syncytial virus (0.05x) 2. Tomato yellow leaf curl China betasatellite (0.37x) 3. Human parainfluenza virus 3 (0.61x)
**Gargles: Lung cancer compared to melanoma**	Higher abundance in Lung cancer	1. ***Fusobacterium*** (2.47x) 2. ***Atopobium*** (2.09x) 3. ***Leptotrichia*** (1.80x) 4. ***[Prevotella]*** (1.77x) 5. ***Porphyromonas*** (1.34x) 6. ***Granulicatella*** (1.07x)	1. ***Neisseria subflava*** (2.05x) 2. ***Prevotella ICM33*** (1.31x)	1. Haemophilus phage HP2 (14.23x) 2. Haemophilus Phage HP1 (7.16x) 3. Human betaherpesvirus 7 (1.83x)
	Lower abundance in Lung cancer	1. ***Neisseria*** (0.49x) 2. ***Actinomyces*** (0.51) 3. ***Rothia*** (0.76x) 4. ***Veillonella*** (0.89x) 5. ***Prevotella*** (0.93x) 6. ***Streptococcus*** (0.98x)	1. ***Rothia dentocariosa*** (0.23x) 2. ***Prevotella melaninogenica* (0.57x)** 3. ***Veillonella dispar*** (0.60x) 4. ***Prevotella pallens*** (0.80x) 5. ***Rothia mucilaginosa*** (0.93x)	N/A
**Lung cancer tumor compared to normal lung tissue**	Higher abundance in Lung tumor	1. *Burkholderia* (1.23x)	N/A	N/A
	Lower abundance in Lung tumor	N/A	N/A	N/A
**Lung cancer lavage compared to lung cancer gargle**	Higher abundance in lung cancer lavage	1. ***Leptotrichia*** (5.46x) 2. *Campylobacter* (2.50x) 3. ***Fusobacterium*** (1.13x) 4. ***Neisseria*** (1.04x)	1. ***Rothia dentocariosa*** (2.71x) 2. ***Porphyromonas* KLE1280** (2.53x) 3. ***Gemella hemolysans*** (1.79x) 4. ***Granulicatella adiacens*** (1.71x) 5.	1. Human parainfluenzavirus 3 (45.8x)
	Lower abundance in lung cancer lavage	1. ***Rothia*** (0.35x) 2. *Atopobium* (0.52x) 3. ***Porphyromona*** *s* (0.54x) 4. ***Streptococcus*** (0.57x) 5. ***Prevotella*** (0.61x) 6. ***Granulicatella*** (0.77x) 7. ***Actinomyces*** (0.78x) 8. ***Veillonella*** (0.93x) 9. ***[Prevotella]*** (0.97x)	1. ***Veillonella dispar*** (0.27x) 2. ***Prevotella pallens*** (0.47x) 3. ***Rothia mucilaginosa*** (0.0.53x) 4. ***Neisseria subflava*** (0.94x)	1. Haemophilus phage HP1 (0.08x) 2. Haemophilus phage HP2 (0.06x) 3. Human betaherpesvirus 7 (0.68x)
**Lung cancer lavage compared to lung tumor**	Higher abundance in lung lavage	1. ***Streptococcus*** (651.67x) 2. ***Prevotella*** (130.63x)	N/A	N/A
	Lower abundance in lung lavage	1. *Burkholderia* (0.08x)	N/A	N/A
**Lung cancer gargle compared to lung tumor**	Higher abundance in lung gargle	3. ***Streptococcus*** (1133.67x) 4. ***Prevotella*** (211.00x)	N/A	N/A
	Lower abundance in lung gargle	1. N/A	N/A	N/A

WGSS, whole genome shotgun sequencing; N/A, Not applicable. Oral commensal bacteria are shown in bold type. Fold changes were calculated as the relative abundance in the case sample divided by the control samples and control sample type one versus control sample type 2.

Lung cancer lavages appeared slightly more diverse than controls, but not significantly (**Table 3**; **Figure 2C**) (Lee et al., 2016; Gomes et al., 2019). Beta diversity, measured by Bray-Curtis dissimilarity, showed substantial differences but no clear separation between cases and controls (**Table 3**; **Figure 2D**).

**Table 3:** Comparison of alpha and beta diversity of bacterial genera, bacterial species, and viral taxa between cases and controls and between sample types among cases.

Comparison	Sequencing methodology					
	16S rRNA gene sequencing		WGSS (bacterial)		WGSS (viral)	
	Alpha diversity (means)	Beta diversity	Alpha diversity (means)	Beta diversity	Alpha diversity (means)	Beta diversity
**Lavages: Lung cancer compared to melanoma**	1. lower 2. lower 3. higher	1. S 2. MS 3. MS	1. higher 2. lower 3. higher	1. NS 2. NS 3. NS	1. higher 2. higher 3. higher	1. NS 2. NS 3. NS
**Gargles: Lung cancer compared to melanoma**	1. lower 2. lower 3. lower	1. NS 2. NS 3. NS	1. lower 2. lower 3. lower	1. NS 2. NS 3. NS	1. higher 2. higher 3. lower	1. NS 2. NS 3. NS
**Lung cancer tumor compared to normal lung tissue**	1. higher 2. equal 3. higher	1. NS 2. NS 3. NS	N/A	N/A	N/A	N/A
**Lung cancer lavage compared to lung cancer gargle**	1. higher 2. higher 3. higher (S)	1. S 2. NS 3. S	1. lower (S) 2. lower 3. lower (S)	1. S 2. NS 3. S	1. lower 2. lower 3. lower (S)	1. S 2. S 3. S
**Lung cancer lavage compared to lung tumor**	1. higher (S) 2. higher (S) 3. higher (S)	1. S 2. S 3. S	N/A	N/A	N/A	N/A
**Lung cancer gargle compared to lung tumor**	1. higher (S) 2. higher (S) 3. higher (S)	**5. **S **6. **S **7. **S	N/A	N/A	N/A	N/A

Metric 1-3 for alpha diversity is Shannon index, Simpson index, and Chao1 index, respectively, and for beta diversity metric 1-3 is Bray-Curtis dissimilarity, weighted UniFrac distance, and unweighted UniFrac distance, respectively. S, significant (p¾0.05), MS, marginally significant (p=0.08 or below); NS, not significant; WGSS, whole genome shotgun sequencing; N/A, Not applicable. Alpha diversity means were compared by T tests (Shannon) and Wilcoxon rank sum tests (Chao1 and Simpson).

##### 3.2.1.2 Whole genome shotgun sequencing

The bacterial species *Granulicatella adiacens* and *Neisseria subflava* were more abundant in cases compared to controls by 6.18 and 15.93-fold, respectively (**Table 2**; **Supplementary Figures S1A, B**). Several species of *Prevotella* were more abundant in controls compared to cases. Alpha diversity estimates revealed no consistent pattern and, along with beta diversity, were not significantly different between cases and controls (**Table 3**; **Supplementary Figures S1C, D**).

The virome of the tracheal lavages was assessed through WGSS sequencing, too, identifying broadly similar prevalence and relative abundance between cases and controls (**Table 2**). Human betaherpesvirus 7 was far more abundant in case versus control lavages but was rare. Though not statistically significant, case lavages consistently showed higher viral alpha diversity than control lavages (**Table 3**). Beta diversity was similar.

#### 3.2.2 Cases vs. controls: oral gargles

##### 3.2.2.1 16S rRNA gene sequencing

**Figure 3:**
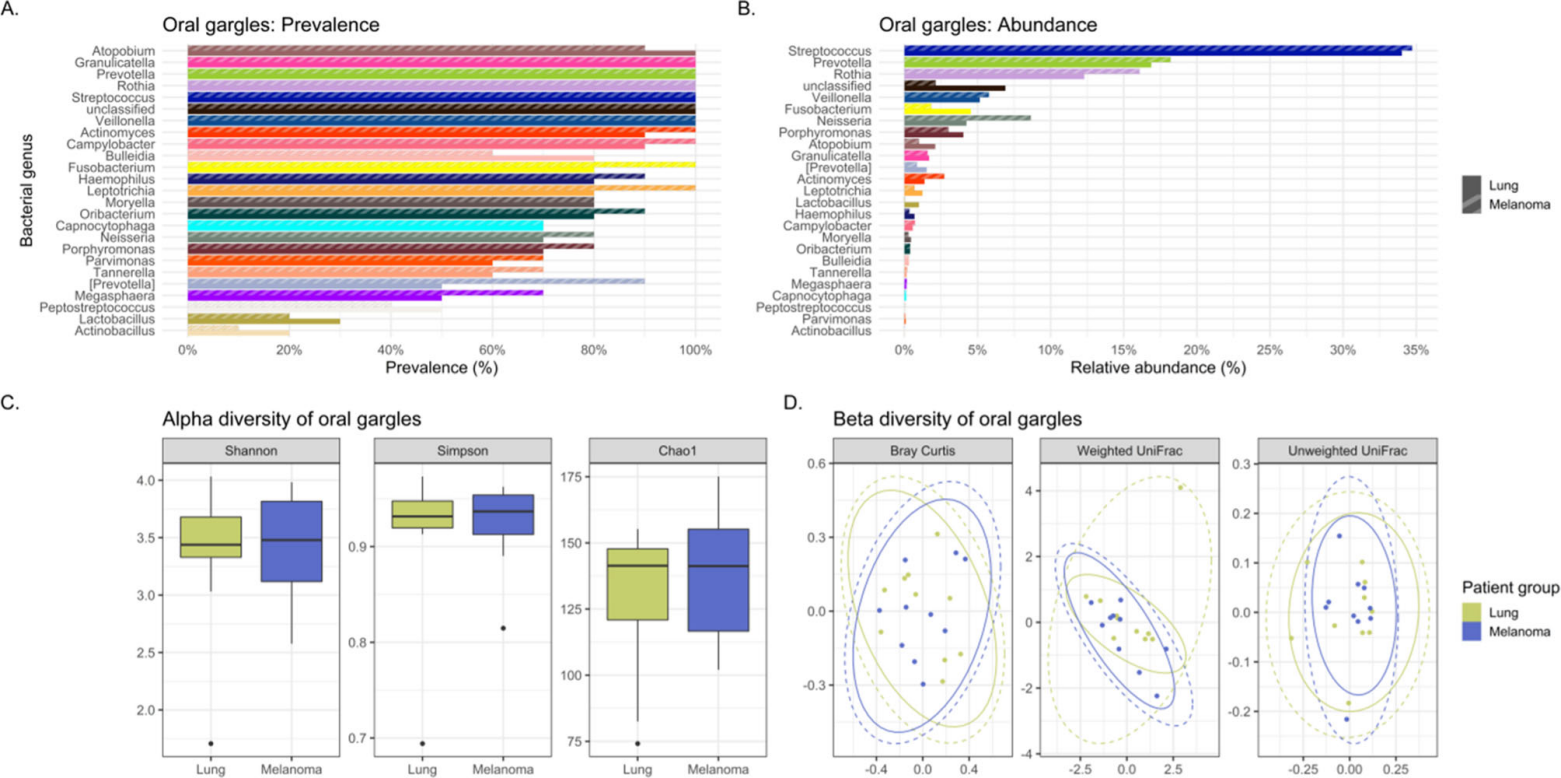
Comparing the bacteriomes, assessed by 16S rRNA gene sequencing, of oral gargles of lung cancer cases and melanoma controls. **(A)** Comparison of the prevalence of bacterial genera in lung cancer versus melanoma control oral gargles. **(B)** Comparison of the relative abundance of bacterial genera in lung cancer versus melanoma control oral gargles. **(C)** Comparison of the alpha diversity, as measured by Shannon, Simpson, and Chao1 indices, between lung cancer case and melanoma control oral gargles. **(D)** Comparison of the beta diversity, measured by Bray Curtis, Weighted and Unweighted UniFrac distance measures, between lung cancer case and melanoma control oral gargles.

Oral gargles from lung cancer and melanoma patients showed very little difference in prevalence (**Figures 1B**, **3A**).

The genus *Prevotella* was more prevalent in controls (90%) compared to cases (50%), while *Granulicatella* was identified in all oral gargles from all patients (**Figure 3A**). Regarding relative abundance, *Streptococcus* and *Prevotella* (Huffnagle et al., 2017) were the most abundant genera in oral gargles from both cases and controls (**Figure 3B**). *Neisseria* was more abundant in controls compared to cases, while *Fusobacterium* was 2.5-fold higher in cases. Alpha and beta diversity showed no significant differences between gargles (**Table 3**; **Figures 3C, D**).

##### 3.2.2.2 Whole genome shotgun sequencing

*Neisseria subflava* was twice as abundant in gargles from lung cancer cases compared to controls, and *Rothia dentocariosa* was slightly more abundant in controls versus cases (**Table 2**; **Supplementary Figure S2B**). Overall, bacterial abundance appeared quite similar among oral gargle samples. Neither alpha nor beta diversity indices demonstrated significant differences between cases and controls (**Table 3**; **Supplementary Figures S2C, D**). There were no significant differences in the case versus control viromes in the gargles (**Supplementary Figure S3**).

#### 3.2.3 Cases: tumor versus normal (non-neoplastic) lung tissue

*16S rRNA gene sequencing:* Considering prevalence, *Propionibacterium*, *Atopobium*, and *Granulicatella* were identified in at least one tumor specimen but not in normal tissues (**Figure 1C**; **Supplementary Figure S4A**). The most abundant genus, albeit rare, in both the tumor and normal tissue was *Burkholderia*, though it is slightly more abundant in tumors (**Table 2**; **Supplementary Figure S4B**). The most abundant in both tissues were unclassified bacteria. Alpha and beta diversity were not significantly different between tissue types (**Table 3**; **Supplementary Figures S4C, D**), though normal tissue generally had lower alpha diversity than tumor tissue (Greathouse et al., 2018).

#### 3.2.4 Cases: lavage versus gargle

##### 3.2.4.1 16S rRNA gene sequencing

Several genera appeared slightly more prevalent in lavages than gargles (**Figure 1D**; **Supplementary Figure S5A**), including *Leptotrichia*, while genera like *Capnocytophaga* were more prevalent in gargles. The genera *Streptococcus*, *Prevotella*, and *Rothia* were more abundant in gargles than lavages, while *Leptotrichia* was >5-fold higher. By the Chao1 index, lavages maintained higher diversity than gargles (**Table 3**; **Supplementary Figure S5C**). Bacterial community structures significantly differed between gargles and lavages by both Bray Curtis dissimilarity and unweighted UniFrac distance (p=0.001) (**Supplementary Figure S5D**).

##### 3.2.4.2 Whole genome shotgun sequencing

*Rothia dentocariosa* was 2.7x more abundant in lavages versus gargles (**Table 2**; **Supplementary Figure S6B**). Interestingly, *E. coli* was not identified in the top 25 most prevalent species of lavages but was observed in all gargle samples. The species *R. mucilaginosa* (28.9% versus 17.9%), *Veillonella dispar* (5.9% versus 1.9%), and two species of *Prevotella* are more abundant in gargles versus lavages, respectively. On the other hand, *Porphyromonas* KLE 1280 (6.5% versus not within the top 25 most abundant species) and *G. adiaciens* (5.1% versus 2.5%) are more abundant in lavages than gargles, respectively (**Supplementary Figure S6B**). Shannon and Chao1 alpha diversity indices revealed gargles to be significantly more diverse compared to lavages (**Table 3**), significantly so by Shannon (p=0.015) and Chao1 (p=0.004) indices. Beta diversity by Bray Curtis dissimilarity (p=0.005) and unweighted UniFrac distance (p=0.002) showed significantly differential bacterial community structures also between lavages and gargles (**Table 3**; **Supplementary Figures S6C, D**).

Considering viral signatures, prevalence appears different between these sample types (**Supplementary Figures S7**) For example, Human parainfluenza virus 3 and respiratory syncytial virus were identified in more lavages (10 and 6, respectively) than gargles (6 and 3, respectively). Human gammaherpesvirus 4 and beta herpesvirus 7 were identified in 5 and 6 gargle samples, but only 1 lavage specimen, respectively. Several non-human, plant and bacterial pathogens were identified in these samples as well. There also appeared to be a much higher proportion of unclassified viral taxa in lung cancer lavages (92.6%) versus gargles (74.6%). Alpha diversity by Chao1 was higher in gargles versus lavages (**Table 3**; **Supplementary Figure S7C**). Beta diversity was significant across Bray Curtis dissimilarity (p=0.001), weighted UniFrac distance (p=0.004), and unweighted UniFrac distance (**Supplementary Figure S7D**).

#### 3.2.5 Cases: lavage versus tumor

*16S rRNA gene sequencing:* The genus *Burkholderia* is more prevalent in tumor tissue compared to lavages (90% versus 40%, respectively) (**Supplementary Figure S8A**). The tumor tissue mostly contained unclassified organisms but did maintain a higher relative abundance of *Burkholderia* than the lavage samples (1.4% versus 0.1%, respectively). However, genera like *Streptococcus*, *Fusobacterium*, *Veillonella*, *Granulicatella, Neisseria*, *Leptotrichia*, *Prevotella*, and *Rothia*, amongst many others, were more abundant in lavages compared to tumor tissues. LEfSe showed that *Burkholderia* and its associated family were significantly differentially abundant in tumors as compared to lavages (data not shown). Many bacterial taxa significantly differentiated lavages from tumors, including *Granulicatella*, *Leptotrichia*, *Neisseria*, *Prevotella*, and *Rothia* (**Supplementary Figures S8A, B**). Alpha diversity was significantly different between lavages and tumor tissue by all three indices, Shannon (p=0.0005), Simpson (p=0.0246), and Chao1 (p=0.0003), whereby intra-sample diversity was consistently higher in lavages versus tumor tissue (**Table 3**; **Supplementary Figure S8C**). Similarly, all three Beta diversity measures showed that bacterial community structure significantly differs between the sample types (p=0.001 across all three indices) (**Supplementary Figure S8D**).

#### 3.2.6 Cases: gargle versus tumor

*16S rRNA gene sequencing:* Most genera were more abundant in gargles versus tumor tissue. A substantially higher number of bacterial taxa were significantly discriminatory between gargles versus tumors, including *Granulicatella*, *Leptotrichia*, *Neisseria*, *Prevotella*, and *Rothia*. Alpha diversity was significantly different between these two sample types for both Simpson (p=0.007) and Chao1 (p=0.043), indicating that oral gargles were more diverse in bacterial species richness and evenness compared to tumor tissue (**Table 3**). Beta diversity significantly differed across all three metrics, showing that these sample types maintain vastly disparate community structures.

## 4 Discussion

By a conservative estimate, there are at least a billion species of bacteria, but only 30,000 are formally named (Dykhuizen, 2005). Less than 2% of those can be cultured and identified in the laboratory (Wade, 2002). However, in the 1980’s, the introduction of the polymerase chain reaction (PCR) targeting the highly conserved ribosomal genes (16S rRNA) of bacteria allowed for the identification of unculturable organisms (Wilson et al., 1997). Subsequent advances in DNA sequencing and other molecular techniques have allowed for inexpensive, rapid culture-independent identification of the vast array of resident microbiota in health (normobiosis or eubiosis) and in disease (dysbiosis), such as cancer. Despite the explosion in microbiome studies primarily in the gut looking for culprit bacteria that may cause malignancies, especially colon cancer and other gastrointestinal diseases, results have been disappointing since findings are inconsistent and not reproducible, likely due to variability of gut microbiota depending upon the sex, race, age, geographic location, and lifestyle factors of diet, exposures, drugs, and exercise (Pierrard and Seront, 2019).

Pierrard and associates and Nyein and associates observed that external factors such as antibiotics appeared to play a vital role in determining the bacterial composition in the gut microbiome which in turn affected immune checkpoint inhibitors (ICI) efficacy in cancer patients (Pierrard and Seront, 2019; Nyein et al., 2022). However, numerous human studies have failed to identify specific species or phyla associated with immunotherapy efficacy in cancer. Other factors, such as bacterial-dependent gut metabolite production that modifies blood metabolites and immune competence, may be essential to ICI efficacy (Tang, 2011). Although the taxonomy of gut microbiota has been under intense investigation to elucidate its role in various cancers, only a few studies have focused on the respiratory microbiome and its relationship to lung cancer. Therefore, we elected to perform this pilot study investigating possible microbial biomarkers indicating the presence of lung cancer and, if positive, then potentially they may be prognostic indicators of the efficacy of cancer treatment.

### 4.1 Oral gargles or tracheal lavage as potential microbial lung cancer biomarkers

#### 4.1.1 Gargles

The primary focus of this study was to evaluate the oral and lower airway microbiome compositions of lung cancer cases compared to melanoma controls to reveal differences with potential applications as biomarkers for lung tumors. Therefore, we compared tracheobronchial lavages and oral gargles collected from lung cancer cases and controls. The results demonstrate that there are few significant differences in the oral gargles between lung cancer and melanoma patients in overall microbial composition using prevalence, abundance, and diversity measures, indicating unfortunately the readily available, noninvasively sampled oral gargle microbiome would *not* likely serve as a lung cancer biomarker.

#### 4.1.2 Tracheal lavages

Beta diversity refers to the variation between the samples of one community (group) compared to another community, such that the microbiome composition of one group with a higher beta diversity indicates a more significant difference from the other group. By 16S rRNA gene sequencing data, beta diversity measured by Bray Curtis dissimilarity demonstrated significant differences (p=0.022) between case and control lavages, indicating that the bacterial communities in lavages from lung cancer versus melanoma were distinct. However, no such trend was observed for gargles. While the lung cancer and control tracheobronchial lavages were significantly different by 16S rRNA-derived beta diversity, it is difficult to say that the lavages will be able to distinguish lung cancer from non-lung cancer patients since WGSS did not clearly replicate these results.

Abundance denotes a specific bacterium’s percentage of a sample’s overall composition. In contrast, prevalence refers to the number (percentage) of cases in a specific group where a bacterium is detected. Significant trends were observed in abundance and prevalence of the lavages. Most noteworthy was *Granulicatella adiacens*, which was more prevalent and abundant in lung cancer cases. *Granulicatella adiacens* is a well-recognized oral commensal bacterium etiologically linked to endocarditis (Cincotta et al., 2015). We found this bacterium as one of the top 25 most abundant genera in lung cancer lavages, and it had a much higher prevalence appearing in virtually *all* lung cancer tracheal lavages (100%) versus only some control lavages (30%), despite being similarly abundant in gargle specimens of both groups. *Granulicatella adiacens* is the same organism that Cameron and associates found in the sputum of lung cancer patients but not controls in a recent pilot study, suggesting this as a potential novel lung cancer biomarker (Cameron et al., 2017). Replication of this finding in our study suggests that this microbe may be important to further investigate as a potential diagnostic biomarker (Cameron et al., 2017) and possibly even a predisposing factor to the development of lung cancer.

Additionally, lavage from the lower airways of our lung cancer cases harbored numerous supraglottic bacteria *Neisseria* (oral commensal), *Capnocytophaga* (oral commensal), *Leptotrichia* (oral commensal) and *Moryella* (oral and intestinal commensal) with twice the prevalence compared to control lavages. *Neisseria subflava*, which commonly colonizes the dorsum of the tongue, was also found in high abundance in lung cancer lavages.

LEfSe (linear discriminant analysis effect size) analysis is used to validate biomarkers by detailing features (bacterial taxa in lavages in this case) that distinguish two groups based on relative abundances. In our study, the LEfSe analysis did show several bacterial taxa, including Fusobacteria and *Neisseria* (especially the oral commensal *N. subflava)* to be significantly 8-fold differentially abundant in the tracheobronchial lavages of lung versus melanoma patients. These intriguing results strongly support continued research into the tracheal microbiota as potential biomarkers of lung cancer, especially the highly prevalent and abundant *Granulicatella adiacens* and *Neisseria subflava*.

### 4.2 Oral gargle or tracheal lavage as proxies of the tumor microbiome

Our study also investigated the potential utility of the oral gargle or tracheobronchial lavage microbiomes as proxies for the tumor microbiome in lung cancer. If the lavage and oral microbiomes were similar to the tumor microbiome, these less invasive sample types could be utilized to study the tumor microbiome more easily. Initially, lavages and gargles were compared to see if the gargle could mimic the lavage microbiota. However, significant differences were found between both bacterial and viral community structures (i.e., beta diversity) and alpha diversity in lavages and gargles. That is, the gargle microbiota were dissimilar from the lavages and cannot be used to represent the lavage microbiota.

Alpha diversity refers to the variation (how diverse it is) of bacteria within a single sample, such that a higher alpha diversity is usually associated with a more diverse, healthier microbiome. In our study, the alpha diversity of lavages versus gargles was likewise different, with gargles consistently maintaining higher bacterial and viral diversity by WGSS. LEfSe, performed on both 16S rRNA gene sequencing and WGSS data, also showed many differentially abundant bacterial taxa and some viral taxa between lavages and gargles. Unfortunately, as a result, these differences prevent oral gargles from acting as clinical proxies for tracheobronchial lavages. Further differences were identified between the tumor, gargles, and lavages, which precludes using these sample types as proxies of one another. This was not surprising, however, considering previous literature that has identified significant differences between lung tissue and oral microbiomes (Yu et al., 2016).

Despite these results, two recent studies have revealed the prognostic biomarker potential of the lung microbiome: one identified associations of the bronchoalveolar lavage microbiome with recurrence (Patnaik et al., 2021), and another identified *Enterobacter* in this same sample type associated with worse survival (Gomes et al., 2019), emphasizing the importance of continued investigation of the lung microbiome in lung cancer. It has already been hypothesized that Enterobacteriaceae, a bacterial family that expresses the common antigen lipopolysaccharide and identified in our study to be significantly more abundant in lavages and tumor tissue versus oral gargles in lung cancer cases, may induce inflammation in lung cancer that could be associated with poor prognosis (Gomes et al., 2019). Other studies have suggested that some microbiota may opportunistically invade damaged lung epithelium caused by smoking and drive tumorigenesis by producing free radicals like ROS/RNS that can damage the TP53 gene (Greathouse et al., 2018). Mouse models further suggest that lung microbiota may contribute to γδ-T cell activation, which are cells that go on to release the cytokines IL-17A and IL-22 (Jin et al., 2019). These cytokines appeared to co-occur with tumor progression in the mice (Jin et al., 2019). Additional studies are needed to provide substantiated evidence of the mechanistic relationships between the microbiome, the immune system, and lung cancer.

### 4.3 The microbiome of tumor and non-neoplastic lung

Differences in the composition of the tumor and normal non-neoplastic tissue microbiomes of lung cancer patients were examined to highlight differences that might suggest a microbial contribution to lung carcinogenesis. If the microbiome signatures differed slightly but maintained similar microbial signatures between tumor and normal tissue, then it might indicate certain microbes from the typical lung environment that could have contributed to tumorigenesis or at least were opportunistic inhabitants of the tumor microenvironment. Indeed, sequencing revealed no significant differences in bacterial relative abundance and alpha or beta diversity between tumor and normal tissue samples. Interestingly, normal tissue had lower alpha diversity than tumor tissue, contrary to previously observed between tumor and healthy tissue controls (Mao et al., 2018). Finally, slight variations in bacterial prevalence were identified: a higher prevalence of the genera *Granulicatella* and *Burkholderia* in tumors was observed, and a higher prevalence of *Neisseria* and *Fusobacterium* in normal tissues.

The genus *Granulicatella*, in particular, has been found in a previous published study to inhabit the tumor microenvironment as it showed “lung cancer stage-specific increases in abundances” (Mur et al., 2018). As it becomes increasingly anaerobic, the production of useful metabolites for this genus increases (Mur et al., 2018). In the current study, *Granulicatella* was also identified as having a higher prevalence not only in tumors and normal tissue but also in tracheobronchial lavages of lung cancer patients versus melanoma controls. Hosgood and associates also found a strong correlation between the finding of *Granulicatella* enriched in lung cancer patients’ oral and sputum samples compared to controls (Hosgood et al., 2014). This provides some intriguing preliminary data suggesting a possible carcinogenic role for some specific bacteria or at least opportunistic inhabitants of the tumor microenvironment, but testing in more extensive cohort studies is needed. The epidemiological relationship of *Granulicatella* may relate to the mechanism of elevated levels of microbial toxins and resultant inflammatory cytokines leading to chronic inflammation-associated carcinogenesis, such as that reported with *M. tuberculosis* and lung cancer (Elinav et al., 2013; Li et al., 2024).

Overall, tracheal lavages and gargles do not provide a consistent microbial signature for the tumor microbiome. Significant differences were observed between the lavage and tumor microbiomes. By all three alpha diversity indices, lavages maintained higher bacterial diversity than tumor tissue, and by all three beta diversity indices, bacterial communities are different between lavages and tumor tissue. LEfSe revealed many bacterial genera that are more abundant in lavages, like *Granulicatella* and *Neisseria*. However, one genus was more abundant in tumor tissue, namely *Burkholderia*, an important Gram-negative pathogen of lung infections in cystic fibrosis patients (Fauroux et al., 2004) and is the causative agent in the life-threatening respiratory illness melioidosis (Wiersinga et al., 2018). Beta diversity indicated significantly different bacterial community structures between oral gargles and tumor tissue. This was not surprising considering previous literature that has identified significant differences between lung tissue and oral microbiomes (Yu et al., 2016). Indices of alpha diversity also showed gargles to be significantly more diverse than tumor tissues.

Given their substantial differences, lavages and gargles cannot accurately represent the tumor microbiome. However, the genus *Burkholderia*, in particular, appeared more abundant and prevalent in tumor tissue than both lavages and gargles, suggesting a potential role in tumorigenesis or at least opportunistic inhabitants of the tumor microenvironment. However, further more mechanistic studies will need to be performed in order to suggest there is a connection between the mere presence of any of these microorganisms and lung carcinogenesis (Li et al., 2024).

### 4.4 Microaspiration

*Silent* microaspiration is the term used to describe the process in people when they have asymptomatic aspiration of small amounts of oropharyngeal or gastric secretions into their tracheobronchial tree and lungs, and it is felt to contribute to the genesis of many lung diseases such as pulmonary fibrosis (Lee et al., 2010). Microaspiration is a common event, occurring in as many as 50% of healthy people (Gleeson et al., 1997), although it is unknown how many have persistent colonization of the tracheobronchial tree with oral commensals. Previous studies by Segal and associates (Segal et al., 2016) demonstrated that the enrichment of oral commensals in the lower airways of normal individuals is associated with increased host inflammatory tone and checkpoint inhibitor markers. Tsay and colleagues found this lower airway dysbiotic signature to distinguish between patients with lung cancer and benign lung nodules (Tsay et al., 2018).

Particularly notable differences in our study are the marked 16-, 6- and 6-fold higher abundance of the oral commensals *Neisseria subflava*, *Granulicatella adiacens*, and *Leptotrichia* in the lung cancer lavages versus controls. Also, the dysbiotic tracheal microbiome had extensive 2-3 times enrichment of oral microbiota (*Granulicatella, Capnocytophaga*, *Leptotrichia, and Neisseria*) in lung cancer patients compared to controls (**Table 2**), perhaps contributing to an inflammatory environment. The control lavages have a markedly reduced abundance of oral taxa, suggesting microaspiration and inflammation occur more significantly in lung cancer patients than in the control lavages. Indeed, beta diversity studies revealed significant differences (*p*=0.022) in bacterial community structures between the lung cancer and the control melanoma lavages.

Patnaik and associates also found oral aspiration as the source of lower airway microbiota in lung cancer, with the actual microbial community in bronchial lavage correlating with lung cancer recurrence after resection (Patnaik et al., 2021). Tsay and colleagues, using RNA-seq analysis of lower airway samples, found that the supraglottic predominant taxa found in the trachea were associated with upregulation of inflammatory pathways for p53 mutation, PI3K/PTEN, ERK and IL6/IL8, such that enrichment of the lower airway with oral commensals may increase local immune tone with upregulation of IL1, IL6, and ERK/MARK, in turn promoting tumor progression, hence suggesting microaspiration may be involved in lung cancer pathogenesis (Tsay et al., 2018).

### 4.5 Microvirome

While the characterization of the human microbiome’s bacterial (and fungal) members, including the respiratory tree, has blossomed in the last decade, studies of the viral component still need to be completed. Many challenges exist with exploration of the microvirome due to its low biomass and enormous number of unclassified species. Even high throughput sequencing technologies are hampered by the small fraction of total DNA in the sample, often present in concentrations too low (< 1% of the reads) to be detected without amplification (Abbas, 2019). Additionally, contaminating human and bacterial DNA and RNA in samples are challenging. Finally, most reads with WGSS are commonly called “viral dark matter” since the results cannot be annotated into taxonomic categories (unclassified) due to the lack of species available in databases (Abbas, 2019). For example, as of 2022, the International Committee on Taxonomy of Viruses listed only 11,273 named viral species (International Committee on Taxonomy of Viruses, 2023) but they estimated there were unknown 100,939,140 viruses, excluding tens of millions of bacteriophage known and unknown species (Racaniello, 2013). As a result, only a relatively small number of viruses were identified in our study, likely because of the low abundance and obvious inability to classify common though unknown viral organisms.

The virome of the tracheal lavages in our study was assessed through WGSS sequencing, which identified broadly similar prevalence and relative abundance between cases and controls among both lavages and gargles. Of the identified viruses, there was a higher abundance of human respiratory syncytial virus in the melanoma versus lung cancer patient lavages. Conversely, human beta-herpesvirus 7 was more abundant in lung cancer lavages versus controls. Oddly, many of the more prevalent viruses we identified in both cases and controls are plant pathogens (e.g., yellow vein viruses and tomato yellow leaf curl viruses), although yellow leaf curl is known to infect tobacco plants, which could conceivably enter the respiratory tree by cigarette smoking.

Viral signatures in the oral gargles demonstrated that bacteriophages targeting *Haemophilus* bacteria were more prevalent in melanoma controls versus lung cancer cases. Human-tropic viruses, such as endogenous retrovirus K and beta-herpesvirus 7, were similarly more prevalent between cases and controls. Although LEfSe identified several unclassified viral signatures as significantly different between cases and controls, neither alpha nor beta diversity indices demonstrated any significant differences between cases and controls. However, unclassified viral signatures were the most abundant in cases and controls.

A comparison of viral signatures between the oral gargle and lavages in the lung cancer cases demonstrated that the prevalence between these two sample types appears different. The most prevalent viral signature in lavages was human parainfluenza virus 3 and the human respiratory syncytial virus compared to gargles. However, human gamma-herpesvirus 4 was identified more commonly in gargles. LEfSe revealed several viral taxa that were significantly differentially abundant between gargles and lavages. Alpha diversity for viral signatures was higher across all three indices in gargles compared to lavages. Finally, beta diversity revealed significant differences in viral community structure between gargles and lavages.

Unfortunately, our WGSS and bioinformatics approaches left most of the viral taxa unclassified in lung cancer lavages (92.6%) and gargles (74.6%), thus hampering meaningful evaluation of the viral microbiome.

### 4.6 Proinflammatory microbiome

Ultimately, the question arises as to why a dysbiotic, inflammatory tracheobronchial microbiome appears to be uniformly associated with lung cancer, and perhaps the answer lies in the multifactorial nature of carcinogenesis as suggested by the human papillomavirus (HPV) and cervical cancer picture. HPV has been convincingly proven to cause 99.7% of cervical cancer (Walboomers et al., 1999). If a woman is found to have high risk HPV on her pelvic exam specimens, then she is at elevated risk for the malignancy, yet at most, only 8% of high risk, HPV-positive women ever develop either pre-cancerous cervical changes or frank cancer (Rodríguez et al., 2010). Recent studies suggest that the primary factor determining the ability of HPV to transform cervical cells is the vaginal microbiota, such that a dysbiotic, inflammatory microbiome is needed. A eubiotic, low diversity, low pH vaginal microbiome, mainly dominated by lactobacillus species, likely helps clear HPV infections and is also cytotoxic by secreting bacteriocins that modulate the immune system to inhibit viral activity. However, the dysbiotic, proinflammatory vaginal microbiome induces oxidative DNA damage and promotes viral transformation of the cervix by the resident HPV (Mitra et al., 2016).

Therefore, we might postulate a similar scenario for the consistent finding of a dysbiotic tracheobronchial microbiome in lung cancer patients. In fact, chronic lung inflammation along with concurrent inflammatory cytokines, growth factors and adhesions molecules provide a favorable environment to promote tumor cell growth and proliferation (Wang et al., 2021). Suppose some or all lung cancer is “caused’ by one or more oncogenic viruses suggested by prior studies, such as HPV (Xiong et al., 2017), bovine leukemia virus (Robinson et al., 2016), and HTLV-1 (Nomori et al., 2011; Robinson et al., 2016). In that case, the development of a dysbiotic, inflammatory tracheobronchial microbiome, such as that found in the current study and others, may be the promoting factor that allows existing colonized, oncogenic viruses to cause malignant transformation in the lung. However, this attractive hypothesis will require many future studies to substantiate.

### 4.7 Limitations and strengths

The small sample size of this study results in significant limitations that may have obscured statistically significant differences in microbiome compositions between lung cancer cases and melanoma controls. The use of melanoma patients undergoing resection of a peripheral extremity tumor under general anesthesia was the best possible proxy for control patients to collected oral gargles and tracheal lavages. Theoretically, the best control would be bronchoscopy and lavages on normal people without cancer or lung disease, but we did not feel ethically comfortable attempting to recruit normal people for a procedure that carries some procedural risk. In addition, the small sample sizes prevent us from appropriate sub-analysis of smokers versus non-smoker results. Future research will require larger cohorts to allow sufficient power to detect clinically meaningful differences that could hold biomarker potential. Although, a more thorough evaluation of specimen contamination could be implemented in future studies, our case and control samples were processed with similar reagents and at the same time, so contamination should not result in a substantial difference in microbiome signatures between our comparison groups.

Due to the study’s case-control design, the effect of changes in the microbiome over time could not be established to identify when microbial alterations may have occurred in lung cancer patients as compared to the controls. Therefore, further research into microbial dysbiosis in lung cancer will ideally require collecting samples at various time points using prospective cohort designs, although this would be a more challenging study to accomplish. However, such a study might enable a better understanding of when microbial dysbiosis occurs and how it is associated with clinically important events, such as disease initiation, progression, or treatment response.

Despite these limitations, this study has several significant strengths, including the direct comparison of the oral, tracheal, lung tumor, and non-neoplastic lung microbiome versus the oral and tracheal microbiome of control patients without lung cancer. Also important is the use of WGSS in addition to 16S rRNA gene sequencing of the specimens. WGSS enabled greater taxonomic resolution, specifically to the species level—more so than 16S rRNA gene sequencing would have enabled alone.

WGSS additionally enabled the elucidation of viral, not merely bacterial, signatures to generate a more holistic view of the microbial environments among the different sample types. However, since the vast majority (93%) of viral signatures were unclassified, we are conducting additional research studies focusing on the more specific PCR approaches targeting specific viral taxa suspected to be associated with lung cancer, including human retroviruses, human papillomavirus (Srinivasan et al., 2009; Xiong et al., 2017), and hepatitis B virus (Sundquist et al., 2014), as documented in our prior pan-microbial array study of biobanked frozen lung cancers (Robinson et al., 2016).

### 4.8 Conclusions

The primary focus of this study was to evaluate the oral and lower airway microbiome compositions of lung cancer cases compared to melanoma controls to reveal any differences that may have potential applications as lung cancer biomarkers. Indeed, in this case-control study, we found that bacterial communities of lung cancer versus melanoma lavages were significantly different although unfortunately no such trend was observed for gargles. Several bacterial taxa, including oral commensals *Neisseria subflava and Granulicatella adiacens* were significantly 8-fold differentially abundant in the tracheobronchial lavages of lung versus melanoma patients suggesting these organisms may warrant future study as more-invasive potential confirmatory biomarkers of lung cancer when bronchoscopy results are equivocal. Like other published studies, we found a dysbiotic tracheal microbiome with extensive 2-3 times enrichment of oral microbiota (higher abundances of oral commensals *Granulicatella, Capnocytophaga*, *Leptotrichia and Neisseria*) in lung cancer patients compared to controls. The control lavages have a markedly reduced abundance of oral taxa. This suggests far more microaspiration and resultant inflammation occurs in lung cancer patients.

The tumor microbiome shows substantial differences between the lavages and gargles, such that they cannot accurately stand in as proxies of the tumor microbiome. However, the genus *Burkholderia* in particular, appeared more abundant and prevalent in tumor tissue versus both lavages and gargles, suggesting a potential role of this organism in tumorigenesis or at least it may be an opportunistic inhabitant of the tumor microenvironment. Finally, for the microvirome, our WGSS and bioinformatics approaches left the vast majority of the viral taxa unclassified in lung cancer and control lavages (92.6%) and in gargles (74.6%), thus hampering a meaningful evaluation of the role of virus in lung cancer. Overall, this study generated encouraging preliminary results confirming some of the findings in the published literature that can be used in hypothesis generation for basing future studies directed at identifying potential microbial biomarkers of lung cancer.

## Data Availability

The original contributions presented in the study are publicly available. This data can be found here: NCBI SRA, PRJNA1177881.

## Supplementary material

Supplementary Figure 1 Comparing the bacteriomes, assessed by whole genome shotgun sequencing, of tracheobronchial lavages from lung cancer cases and melanoma controls. **(A)** Comparison of the prevalence of bacterial species. **(B)** Comparison of the relative abundance of bacterial species. **(C)** Comparison of the alpha diversity, as measured by Shannon, Simpson, and Chao1 indices. **(D)** Comparison of the beta diversity, measured by Bray Curtis, Weighted and Unweighted UniFrac distance measures.

Supplementary Figure 2 Comparing the bacteriomes, assessed by whole genome shotgun sequencing, of oral gargles of lung cancer cases and melanoma controls. **(A)** Comparison of the prevalence of bacterial species in lung cancer versus melanoma control oral gargles. **(B)** Comparison of the relative abundance of bacterial species in lung cancer versus melanoma control oral gargles. **(C)** Comparison of the alpha diversity, as measured by Shannon, Simpson, and Chao1 indices, between lung cancer case and melanoma control oral gargles. **(D)** Comparison of the beta diversity, measured by Bray Curtis, Weighted and Unweighted UniFrac distance measures, between lung cancer case and melanoma control oral gargles.

Supplementary Figure 3 Comparing the viromes, assessed by whole genome shotgun sequencing, of oral gargles of lung cancer cases and melanoma controls. **(A)** Comparison of the prevalence of viral species in lung cancer versus melanoma control oral gargles. **(B)** Comparison of the relative abundance of viral species in lung cancer versus melanoma control oral gargles. **(C)** Comparison of the alpha diversity, as measured by Shannon, Simpson, and Chao1 indices, between lung cancer case and melanoma control oral gargles. **(D)** Comparison of the beta diversity, measured by Bray Curtis, Weighted and Unweighted UniFrac distance measures, between lung cancer case and melanoma control oral gargles.

Supplementary Figure 4 Comparing the bacteriomes, assessed by 16S rRNA gene sequencing, of tumor and normal tissue from lung cancer patients. **(A)** Comparison of the prevalence of bacterial genera in lung cancer tumor versus normal tissue. **(B)** Comparison of the relative abundance of bacterial genera in lung cancer tumor versus normal tissue. **(C)** Comparison of the alpha diversity, as measured by Shannon, Simpson, and Chao1 indices, between tumor and normal tissue from lung cancer patients. **(D)** Comparison of the beta diversity, measured by Bray Curtis, Weighted and Unweighted UniFrac distance measures, between tumor and normal tissue from lung cancer patients.

Supplementary Figure 5 Comparing the bacteriomes, assessed by 16S rRNA gene sequencing, of TBLs and oral gargles of lung cancer cases. **(A)** Comparison of the prevalence of bacterial genera in lung cancer oral gargles versus TBLs. **(B)** Comparison of the relative abundance of bacterial genera in lung cancer versus melanoma control oral gargles. **(C)** Comparison of the alpha diversity, as measured by Shannon, Simpson, and Chao1 indices, between lung cancer oral gargles and TBLs. **(D)** Comparison of the beta diversity, measured by Bray Curtis, Weighted and Unweighted UniFrac distance measures, between lung cancer oral gargles and TBLs.

Supplementary Figure 6 Comparing the bacteriomes, assessed by whole genome shotgun sequencing, of TBLs and oral gargles of lung cancer cases. **(A)** Comparison of the prevalence of bacterial species in lung cancer oral gargles versus TBLs. **(B)** Comparison of the relative abundance of bacterial species in lung cancer oral gargles versus TBLs. **(C)** Comparison of the alpha diversity, as measured by Shannon, Simpson, and Chao1 indices, between lung cancer oral gargles and TBLs. **(D)** Comparison of the beta diversity, measured by Bray Curtis, Weighted and Unweighted UniFrac distance measures, between lung cancer oral gargles and TBLs.

Supplementary Figure 7 Comparing the viromes, assessed by whole genome shotgun sequencing, of oral gargles and TBLs of lung cancer cases. **(A)** Comparison of the prevalence of viral species in lung cancer oral gargles versus TBLs. **(B)** Comparison of the relative abundance of viral species in lung cancer oral gargles versus TBLs. **(C)** Comparison of the alpha diversity, as measured by Shannon, Simpson, and Chao1 indices, between lung cancer oral gargles and TBLs. **(D)** Comparison of the beta diversity, measured by Bray Curtis, Weighted and Unweighted UniFrac distance measures, between lung cancer oral gargles and TBLs.

Supplementary Figure 8 Comparing the bacteriomes, assessed by 16S rRNA gene sequencing, of TBLs and tumor tissue of lung cancer cases. **(A)** Comparison of the prevalence of bacterial genera in lung cancer TBLs versus tumor tissue. **(B)** Comparison of the relative abundance of bacterial genera in lung cancer TBLs versus tumor tissue. **(C)** Comparison of the alpha diversity, as measured by Shannon, Simpson, and Chao1 indices, between lung cancer TBLs versus tumor tissue. **(D)** Comparison of the beta diversity, measured by Bray Curtis, Weighted and Unweighted UniFrac distance measures, between lung cancer TBLs versus tumor tissue.
