# Supplementary figures and images for: Metagenomic characterization of the tracheobronchial microbiome in lung cancer
Source: Front Microbiomes. 2024 Nov 21;3:1457537. doi: 10.3389/frmbi.2024.1457537 (PMC12993488; doi:10.3389/frmbi.2024.1457537)

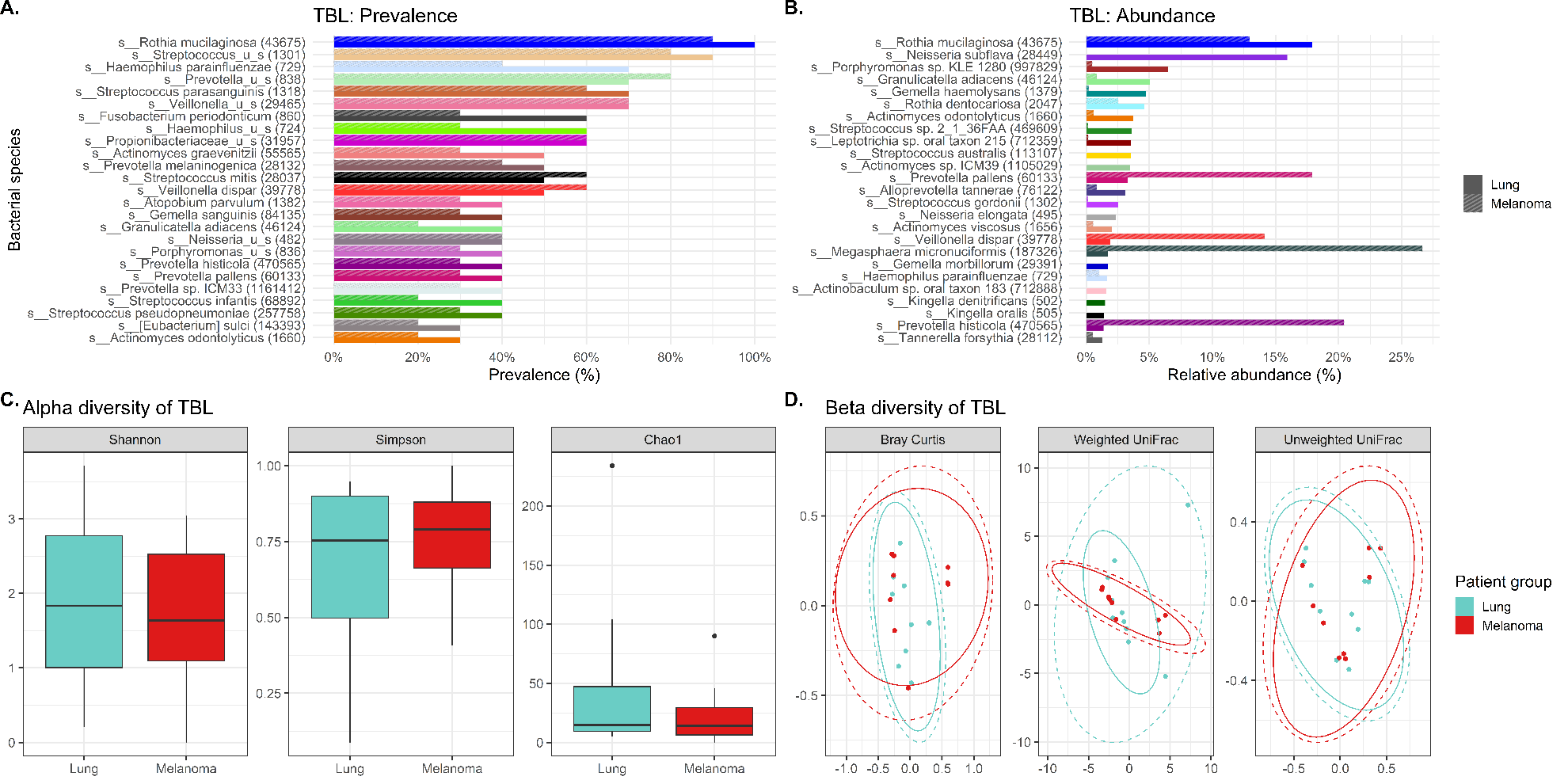

Supplement: Supplementary Figure 1 — Comparing the bacteriomes, assessed by whole genome shotgun sequencing, of tracheobronchial lavages from lung cancer cases and melanoma controls. (A) Comparison of the prevalence of bacterial species. (B) Comparison of the relative abundance of bacterial species. (C) Comparison of the alpha diversity, as measured by Shannon, Simpson, and Chao1 indices. (D) Comparison of the beta diversity, measured by Bray Curtis, Weighted and Unweighted UniFrac distance measures. [file Image1.tiff]

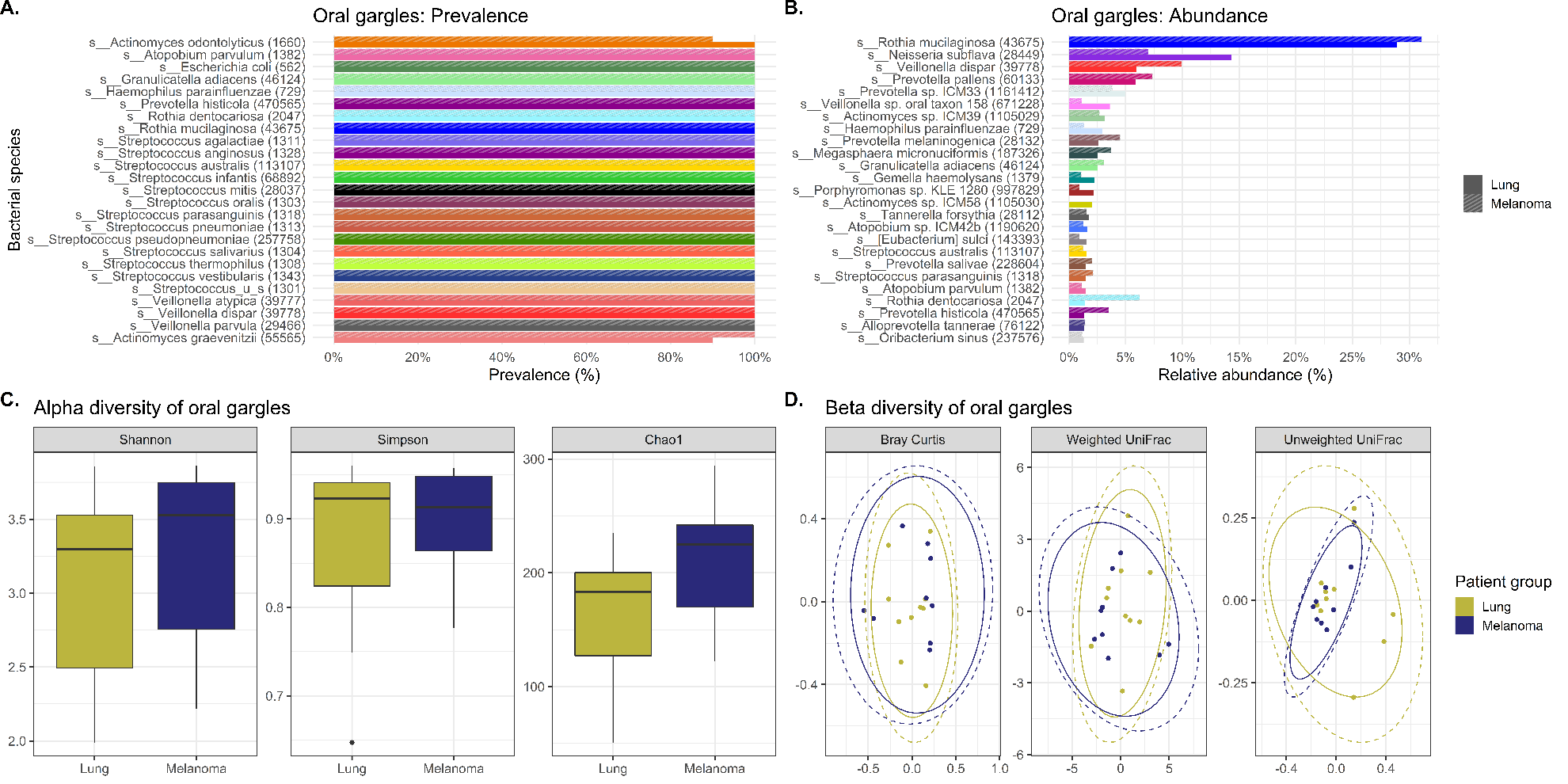

Supplement: Supplementary Figure 2 — Comparing the bacteriomes, assessed by whole genome shotgun sequencing, of oral gargles of lung cancer cases and melanoma controls. (A) Comparison of the prevalence of bacterial species in lung cancer versus melanoma control oral gargles. (B) Comparison of the relative abundance of bacterial species in lung cancer versus melanoma control oral gargles. (C) Comparison of the alpha diversity, as measured by Shannon, Simpson, and Chao1 indices, between lung cancer case and melanoma control oral gargles. (D) Comparison of the beta diversity, measured by Bray Curtis, Weighted and Unweighted UniFrac distance measures, between lung cancer case and melanoma control oral gargles. [file Image2.tiff]

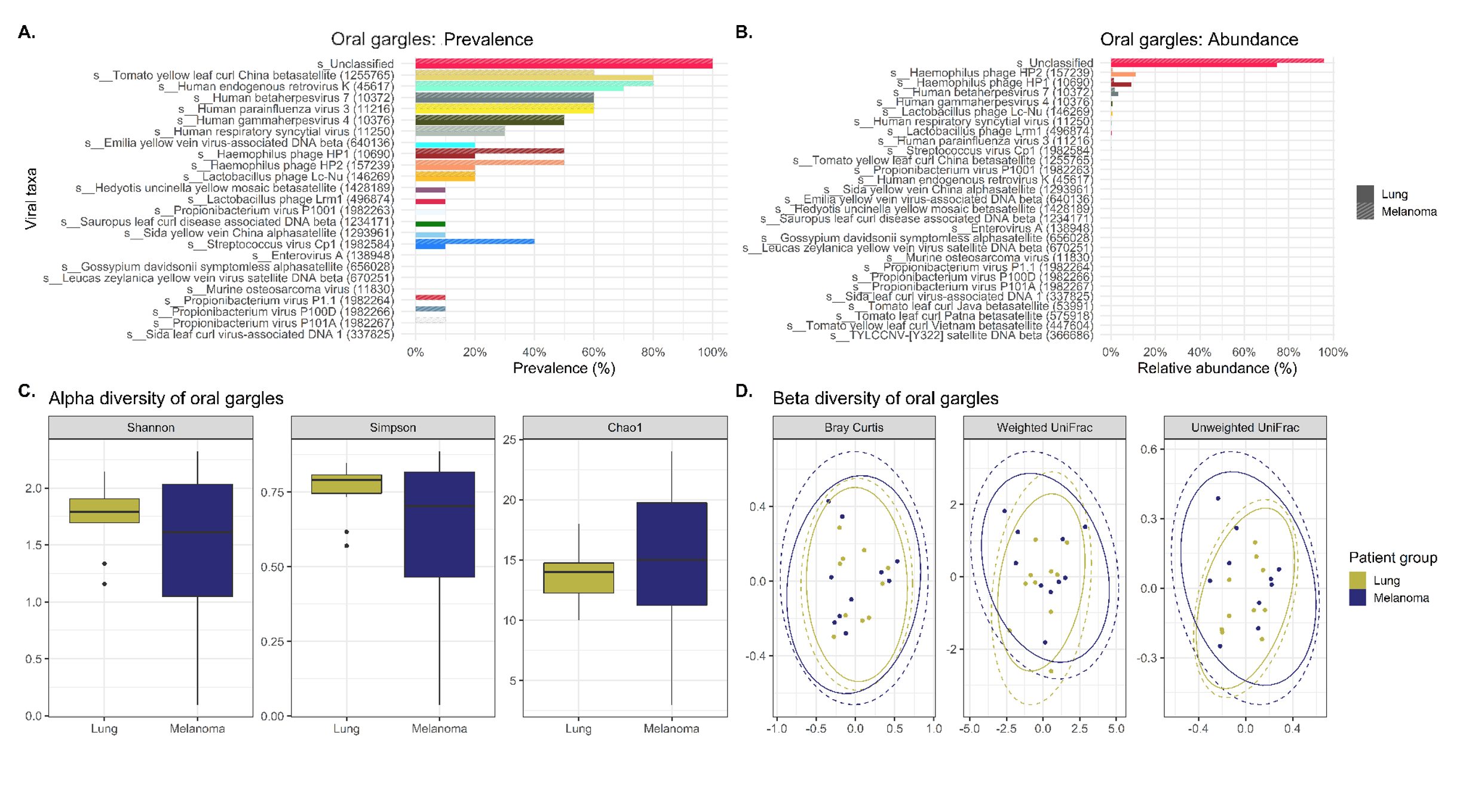

Supplement: Supplementary Figure 3 — Comparing the viromes, assessed by whole genome shotgun sequencing, of oral gargles of lung cancer cases and melanoma controls. (A) Comparison of the prevalence of viral species in lung cancer versus melanoma control oral gargles. (B) Comparison of the relative abundance of viral species in lung cancer versus melanoma control oral gargles. (C) Comparison of the alpha diversity, as measured by Shannon, Simpson, and Chao1 indices, between lung cancer case and melanoma control oral gargles. (D) Comparison of the beta diversity, measured by Bray Curtis, Weighted and Unweighted UniFrac distance measures, between lung cancer case and melanoma control oral gargles. [file Image3.tiff]

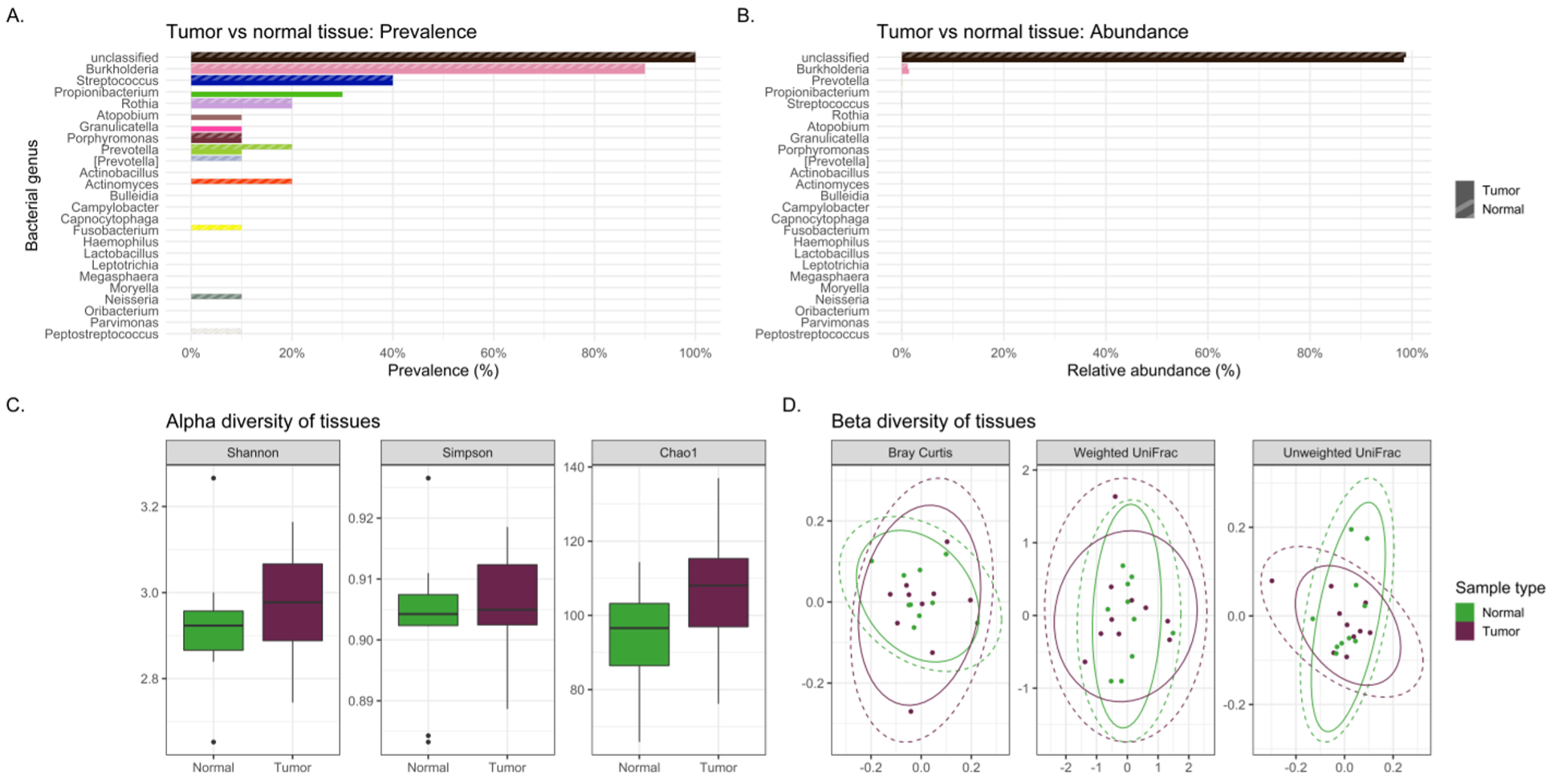

Supplement: Supplementary Figure 4 — Comparing the bacteriomes, assessed by 16S rRNA gene sequencing, of tumor and normal tissue from lung cancer patients. (A) Comparison of the prevalence of bacterial genera in lung cancer tumor versus normal tissue. (B) Comparison of the relative abundance of bacterial genera in lung cancer tumor versus normal tissue. (C) Comparison of the alpha diversity, as measured by Shannon, Simpson, and Chao1 indices, between tumor and normal tissue from lung cancer patients. (D) Comparison of the beta diversity, measured by Bray Curtis, Weighted and Unweighted UniFrac distance measures, between tumor and normal tissue from lung cancer patients. [file Image4.tiff]

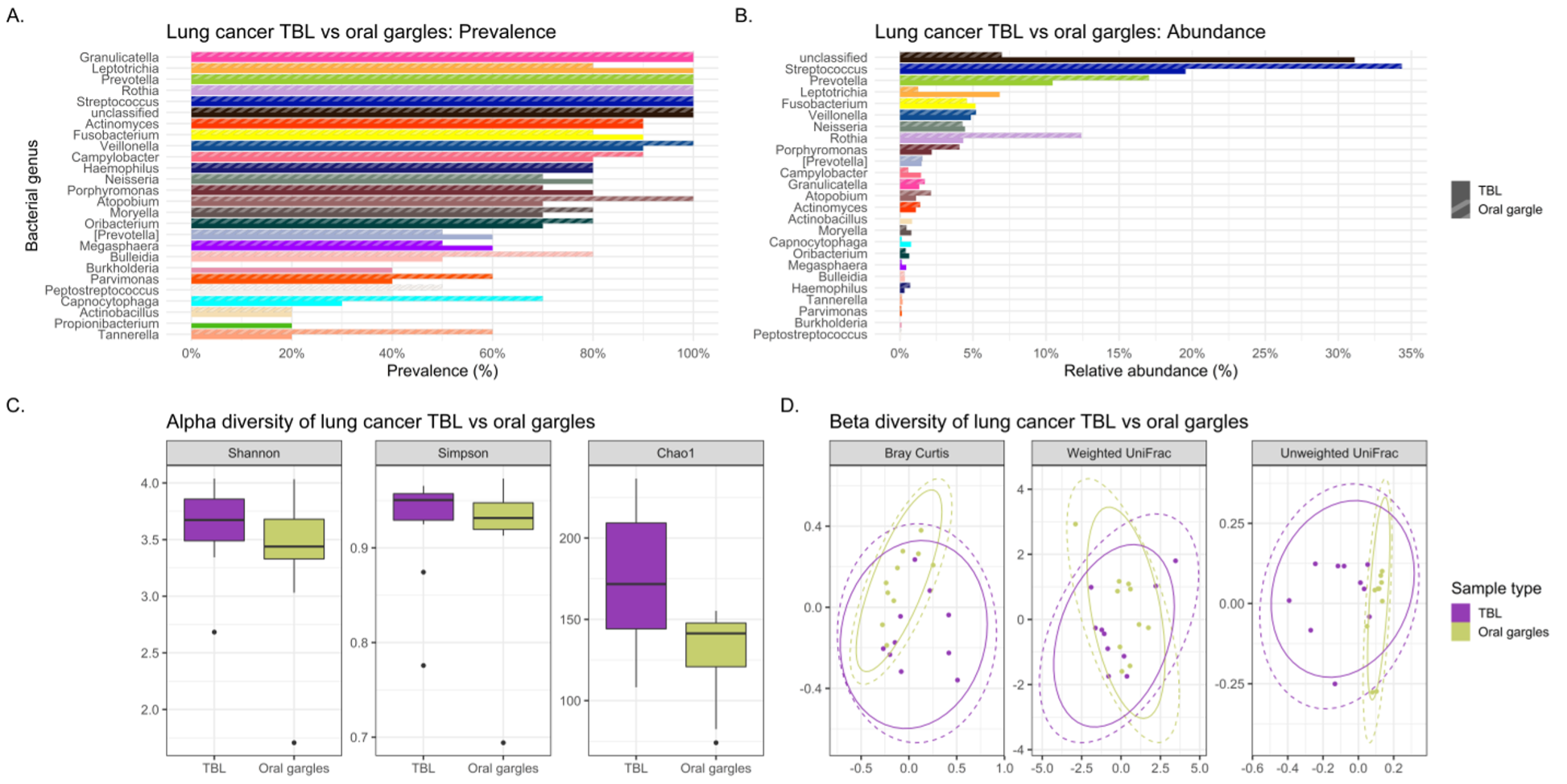

Supplement: Supplementary Figure 5 — Comparing the bacteriomes, assessed by 16S rRNA gene sequencing, of TBLs and oral gargles of lung cancer cases. (A) Comparison of the prevalence of bacterial genera in lung cancer oral gargles versus TBLs. (B) Comparison of the relative abundance of bacterial genera in lung cancer versus melanoma control oral gargles. (C) Comparison of the alpha diversity, as measured by Shannon, Simpson, and Chao1 indices, between lung cancer oral gargles and TBLs. (D) Comparison of the beta diversity, measured by Bray Curtis, Weighted and Unweighted UniFrac distance measures, between lung cancer oral gargles and TBLs. [file Image5.tiff]

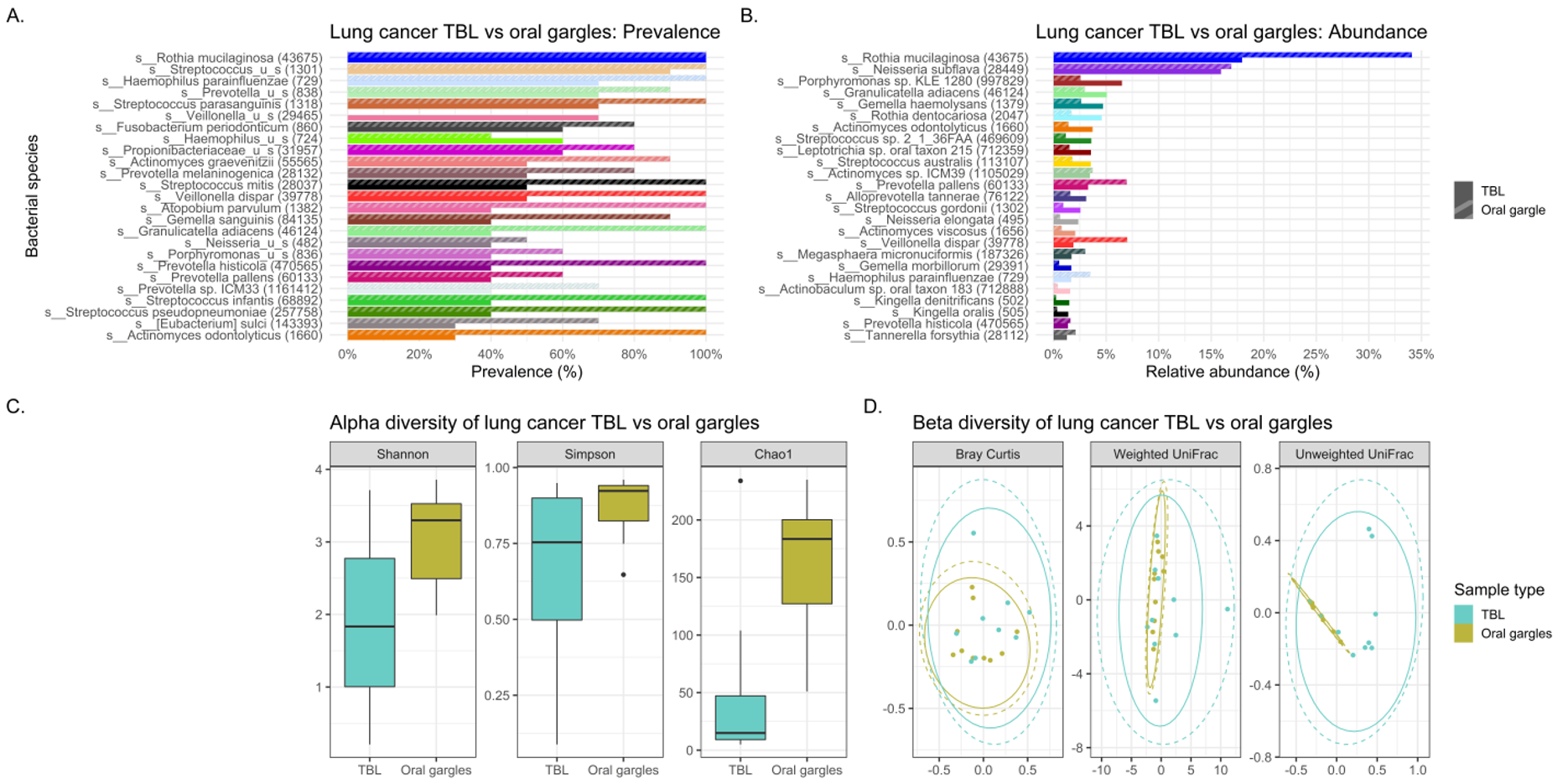

Supplement: Supplementary Figure 6 — Comparing the bacteriomes, assessed by whole genome shotgun sequencing, of TBLs and oral gargles of lung cancer cases. (A) Comparison of the prevalence of bacterial species in lung cancer oral gargles versus TBLs. (B) Comparison of the relative abundance of bacterial species in lung cancer oral gargles versus TBLs. (C) Comparison of the alpha diversity, as measured by Shannon, Simpson, and Chao1 indices, between lung cancer oral gargles and TBLs. (D) Comparison of the beta diversity, measured by Bray Curtis, Weighted and Unweighted UniFrac distance measures, between lung cancer oral gargles and TBLs. [file Image6.tiff]

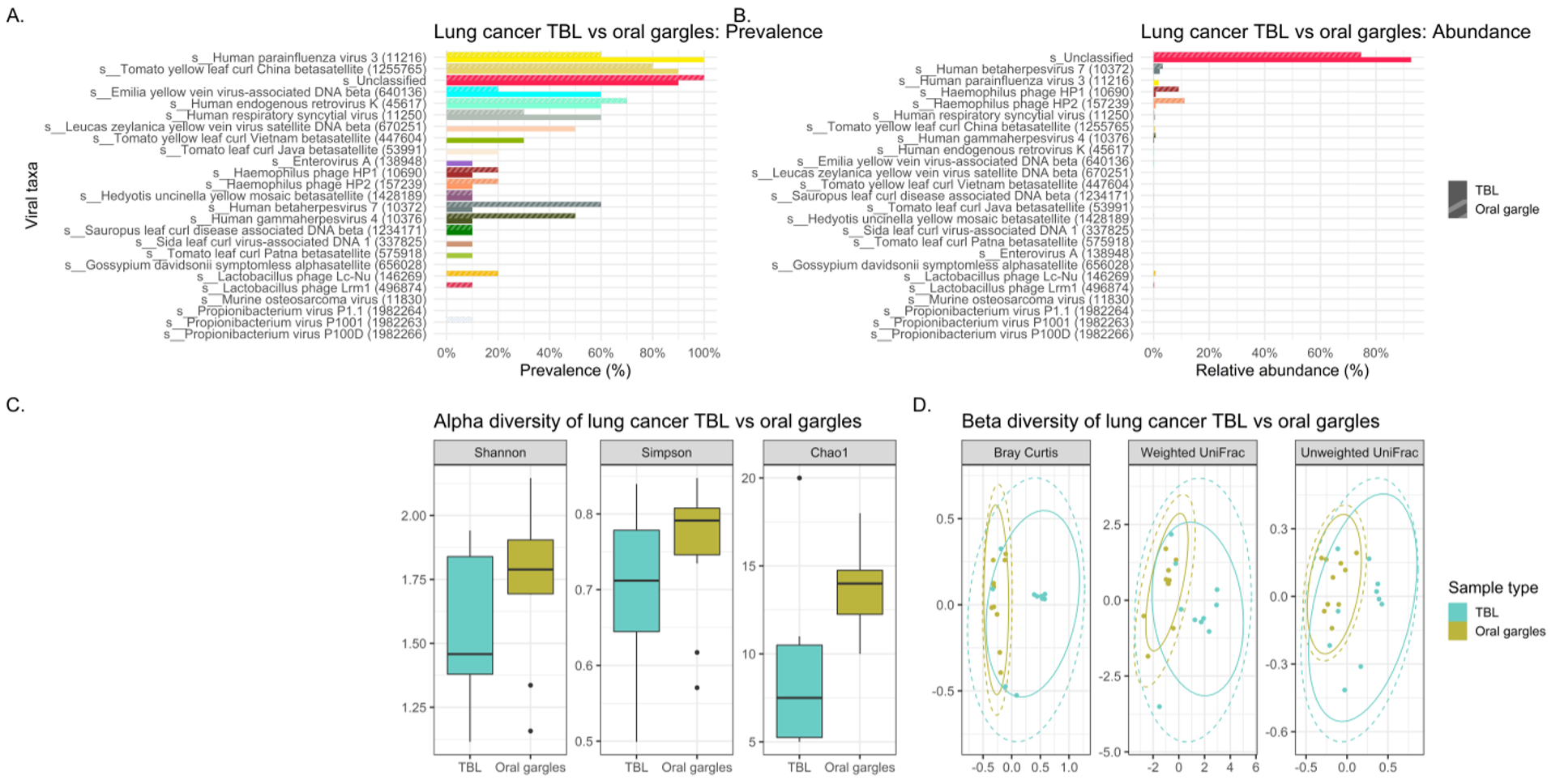

Supplement: Supplementary Figure 7 — Comparing the viromes, assessed by whole genome shotgun sequencing, of oral gargles and TBLs of lung cancer cases. (A) Comparison of the prevalence of viral species in lung cancer oral gargles versus TBLs. (B) Comparison of the relative abundance of viral species in lung cancer oral gargles versus TBLs. (C) Comparison of the alpha diversity, as measured by Shannon, Simpson, and Chao1 indices, between lung cancer oral gargles and TBLs. (D) Comparison of the beta diversity, measured by Bray Curtis, Weighted and Unweighted UniFrac distance measures, between lung cancer oral gargles and TBLs. [file Image7.tiff]

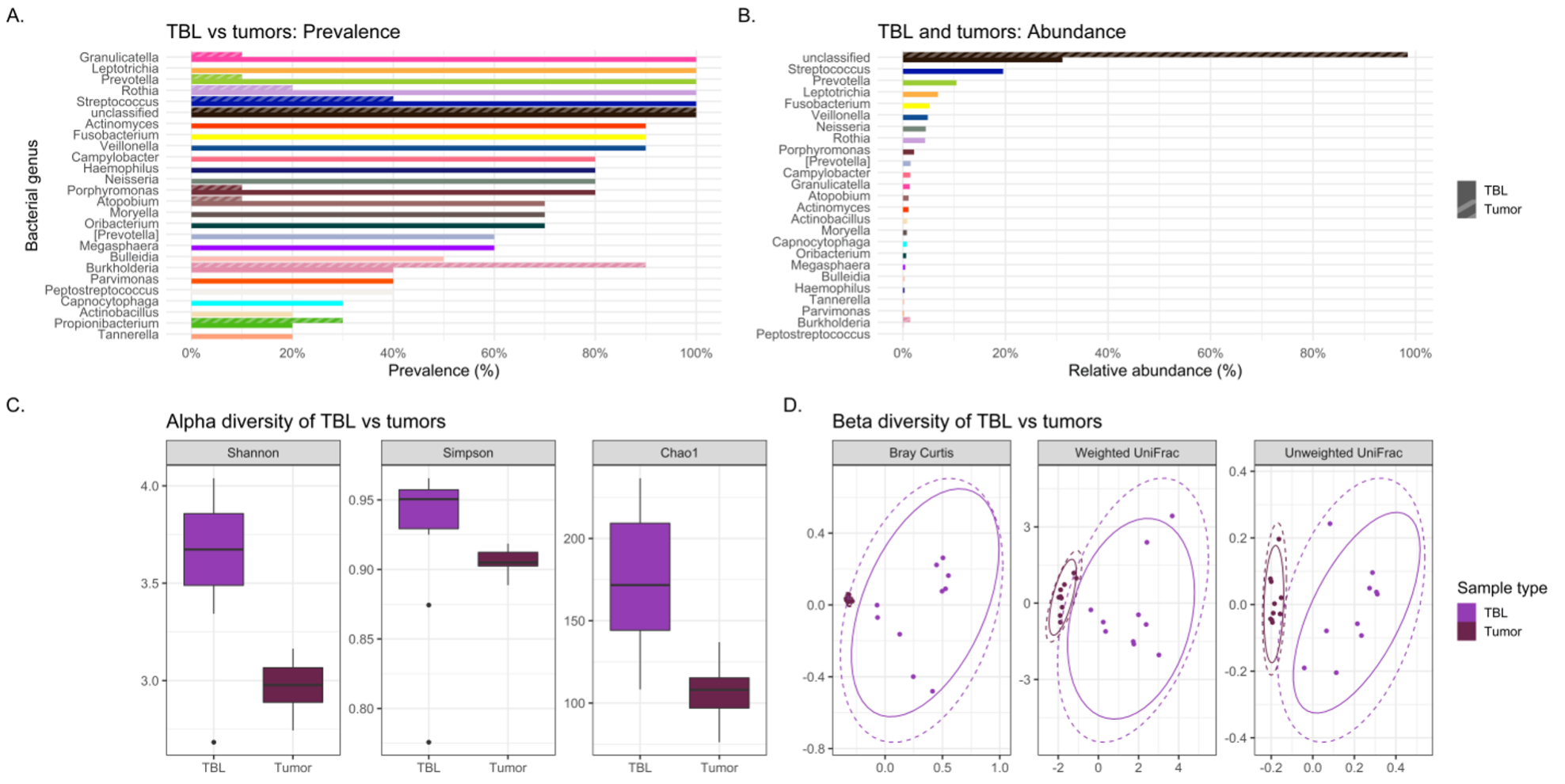

Supplement: Supplementary Figure 8 — Comparing the bacteriomes, assessed by 16S rRNA gene sequencing, of TBLs and tumor tissue of lung cancer cases. (A) Comparison of the prevalence of bacterial genera in lung cancer TBLs versus tumor tissue. (B) Comparison of the relative abundance of bacterial genera in lung cancer TBLs versus tumor tissue. (C) Comparison of the alpha diversity, as measured by Shannon, Simpson, and Chao1 indices, between lung cancer TBLs versus tumor tissue. (D) Comparison of the beta diversity, measured by Bray Curtis, Weighted and Unweighted UniFrac distance measures, between lung cancer TBLs versus tumor tissue. [file Image8.tiff]
